# Negative shocks predict change in cognitive function and preferences: assessing the negative affect and stress hypothesis

**DOI:** 10.1038/s41598-021-83089-0

**Published:** 2021-02-11

**Authors:** Francesco Bogliacino, Cristiano Codagnone, Felipe Montealegre, Frans Folkvord, Camilo Gómez, Rafael Charris, Giovanni Liva, Francisco Lupiáñez-Villanueva, Giuseppe A. Veltri

**Affiliations:** 1 Facultad de Ciencias Económicas, Universidad Nacional de Colombia, Kr 30, No 45-03, Bogotá, Colombia; 2 Centro de Investigaciones Para El Desarrollo, Bogotá, Colombia; 3 Università Degli Studi Di Milano, Milan, Italy; 4 Open Evidence Research, Milan, Italy; 5 Universitat Oberta de Catalunya, Barcelona, Spain; 6 Tillburg School of Humanities and Digital Sciences, Tilburg University, Tilburg, The Netherlands; 7 Economic Science Institute, Chapman University, Orange County, CA USA; 8 Università Degli Studi Di Trento, Trento, Italy

**Keywords:** Cognitive control, Cooperation, Decision

## Abstract

In the context of the current COVID-19 pandemic, households throughout the world have to cope with negative shocks. Previous research has shown that negative shocks impair cognitive function and change risk, time and social preferences. In this study, we analyze the results of a longitudinal multi-country survey conducted in Italy (N = 1652), Spain (N = 1660) and the United Kingdom (N = 1578). We measure cognitive function using the Cognitive Reflection Test and preferences traits (risk, time and social preferences) using an experimentally validated set of questions to assess the differences between people exposed to a shock compared to the rest of the sample. We measure four possible types of shocks: labor market shock, health shock, occurrence of stressful events, and mental health shock. Additionally, we randomly assign participants to groups with either a recall of negative events (more specifically, a mild reinforcement of stress or of fear/anxiety), or to a control group (to recall neutral or joyful memories), in order to assess whether or not stress and negative emotions drive a change in preferences. Results show that people affected by shocks performed worse in terms of cognitive functioning, are more risk loving, and are more prone to punish others (negative reciprocity). Data do not support the hypotheses that the result is driven by stress or by negative emotions.

## Introduction

As a consequence of the current COVID-19 pandemic, an enormous number of households around the globe have been negatively hit by different shocks; either health related, as a result of the disease itself or as an obligation to postpone treatments, or economically related, due to the cost of the various mitigation strategies in terms of shutdowns, layoffs and the exit of firms^1,2^. Negative shocks are defined as losses of income or accumulated assets and can be classified according to the source of the loss; namely, health, labor market, natural disaster, and poverty, amongst others.

This study analyzes the relationship between having suffered negative shocks and individual cognitive functioning and preferences, including time and risk preferences (i.e., risk aversion and time discounting), and social preferences. Cognitive function is defined as the capacity of an individual to solve tasks for which relying on intuition and instinct is not a reliable guide and so reflection is needed. These tasks include reasoning (fluid intelligence), working memory (storing and processing information) and cognitive control (the ability to inhibit external or internal stimuli from distracting, and the flexibility to move from one task to another)^3,4^. Risk aversion is defined as the willingness to accept certain amounts in exchange for lotteries for which the expected value is larger^5,6^. Furthermore, time discounting is a measure of the willingness to give up something today in exchange for a larger sum tomorrow^7^. Finally, social preference means showing other-regarding concerns when it is costly and does not depend upon strategic considerations: examples are altruism and reciprocity in distributive choices, following social norms, participating in the community, and partaking in cooperative behavior in social interactions^8^.

Decision making depends on preferences, beliefs, and constraints, and occurs using scarce cognitive resources to process information to produce actual choices^9,10^. As a result, cognitive function and preferences are of the utmost importance to understand how having suffered shocks changed which decisions are being taken regarding searching for jobs, the management of savings, consumption activities, collective actions, etc., and the aggregate implications of the pandemic and the lockdown for our societies and our economies. In particular, understanding a change in cognitive function makes it possible to predict the likelihood of making suboptimal decisions; a change in risk preferences allows predicting decisions under uncertain scenarios; time discounting guides us in the understanding of savings and other intertemporal decisions (e.g., education); and finally, social preferences help us to anticipate how households and persons will trust, cooperate, donate, and in general, participate in the life of the community.

The previous literature has studied negative shocks because they are experienced during the course of a lifetime for a variety of reasons; for example, because of macroeconomic volatility or natural events^11–14^. These shocks are usually mild, and to some extent more expected and perceived as part of life, because modern economies have developed a variety of market and social policy mechanisms to insure an individual’s consequences from these shocks, such as unemployment subsidies. Shocks have also been studied in developing countries where poverty and violence are widespread, and both are associated with shocks^15–18^, where the institutional environment is less capable of absorbing these shocks and instead leaves households at their mercy. More generally, both developing and developed countries are less resilient to aggregate and correlated shocks (shocks that hit many households at the same time and in the same direction), in which case market insurance mechanisms are absent and the government may face financing constraints. This is a further reason to investigate the consequences of the situation that is currently unfolding due to the COVID-19 pandemic and the various mitigation strategies in place.

Studies have shown that economic shocks have a negative impact on cognitive function and a positive impact on time discounting, risk aversion and social preferences. To begin with the effect of shocks on cognitive function, some of the evidence comes from laboratory experiments where shocks are induced as losses over a Real Effort Task^19^, and where shocks are measured as large income windfalls for sugar cane farmers in rural India^16^. No effect was found for paycheck natural variations^20^, although in the latter case the shock is temporary, expected, and expected to be temporary.

Time discounting and risk aversion are also increased when people face losses in controlled laboratory experiments^21^. Decker and Schimtz^22^ find a similar result for health shock and risk aversion, although in this case the authors rely on matching over observables characteristics and causal interpretation is less credible. Voors et al^23^ document increasing time discounting, but more risk-loving behaviors in the case of negative shocks associated with violence in Burundi, using an instrumental variable strategy.

Finally, social preferences have been studied either in a class of standard strategic interactions (e.g., trust games, gift exchange, ultimatum game, dictator game), where a subset of participants face plausible naturally occurring exogenous shocks or in quasi-experimental studies where partaking in the community, electoral participation and other community involvement is studied in situations in which there is a gradient in terms of exposure to shocks. Studies on the effects of natural disasters show an increase in pro-sociality^24,25^, and the literature on conflict related violence finds an increase in pro-sociality^26^ and an increase in reciprocity^27^. One study has shown that when pure endowment shocks are induced in a two-person trust game, people become less pro-social, however in this case, inequality is more salient than shock because the focus is on the comparison of the endowments between the two counterparts and arguably the latter (rather than the former) is driving the results^28^. Other studies have shown that anti-social behavior after negative shocks occur variously, due to rainfall variations in Germany^29^, grapevine diseases in nineteenth century France^30^, the collapse of financial schemes^31^, and trade shocks^32^; with the finding that under weak institutions, shocks drive anti-social behavior. This latter strand seems to be at odds with the rest of the literature and will be addressed in the Discussion.

Plausible theoretical explanations for the cognitive impact of negative shocks include scarcity, stress, and negative emotions. According to the scarcity hypothesis, negative shocks tax mental bandwidth, reducing cognitive control and fluid intelligence. According to an alternative hypothesis, stress (i.e., the condition by which environmental demands exceed the regulatory capacity of the body) decreases performance according to evidence from mammals^33^ due to alterations in the neuroendocrine network that may affect the prefrontal cortex^34,35^. For the third hypothesis, it is assumed that negative emotions operate through a similar mechanism^15^, in particular negative emotions activate the amygdala that may trigger a number of automatic reactions overcoming cognitive control and fluid intelligence.

For the impact of shocks on risk and time preferences, Haushofer and Fehr^36^ reviewed the literature on negative affect, stress, risk aversion, and time discounting. In the included studies, stress and negative affect were administered in placebo-controlled experiments through fear induction, minor electric shocks, or the supply of hydrocortisone. The results showed that these negative shocks increased time discounting and risk aversion (the result is not replicated in Kassas et al^37^, though). A correlational study by Nguyen and Noussair^38^ showed that negative emotions are associated with risk aversion. Since stress and negative affect are increased by negative shocks, stress and negative emotions are plausible mechanisms to explain why exposure to shock may change time and risk preferences.

Finally, to search for plausible explanations for the effect of negative shocks on social preferences, we can look to the literature that has documented an increase in pro-social behavior after exposure to violence related shocks (reviewed in Bauer et al^26^). It is argued that this effect may be due to a reassessment of individual beliefs (in particular, the importance of social capital in uncertain environments, where the rentability of other forms of capital is severely affected) or aspirations (*post shock growth*), or to evolutionary mechanisms that reward in-group cooperation in intra-groups conflicts—however, the latter is mainly in relation to conflict related shocks and may be less relevant when the exposure is at the individual level (Bauer et al^26^). Nevertheless, Bogliacino, Gómez, and Grimalda^39^ have assessed these mechanisms and found no support for them. They have thus speculated that negative emotions may be the driving force in these situations, either because they impair cognitive performance, indirectly inducing more pro-sociality (Social Heuristics Hypothesis^40^), or they may directly trigger pro-sociality because the warm glow of giving undoes the unpleasant negative emotions themselves^41^. This hypothesis is consistent with evidence from Bosman and Widen^42^ and Joffily et al^43^, although both studies are correlational and not causal.

In this article, we present the results of a survey that we have conducted in order to study the effects of exposure to COVID-19 and the state of lockdown in Italy, Spain, and the United Kingdom (UK), where a representative sample of the same participants has been followed starting from 24 April 2020, answering two questionnaires separated by one week (defined as wave one and wave two). In this paper, we present the results of these subjects who have answered wave two of the study, and for whom we also have the data from the baseline survey conducted in wave one (starting on 24 April 2020). During both wave one and wave two, we have collected information on the exposure to shocks. In wave two, we have measured cognitive performance, and a set of preference traits (risk and time preferences, altruism, trust, positive and negative reciprocity) to assess whether or not they differ in people affected by negative shocks compared with people who were not affected. Additionally, by randomly assigning participants to a negative recall task, mildly reinforcing stress and negative emotions with respect to control recall (recalling neutral or joyful experiences), we use a difference-in-difference approach to assess to what extent, stress and negative affect may cause a change in cognitive functioning and preferences. Although this is a survey with unincentivized responses, both the instrument used to measure cognitive performance (the Cognitive Reflection Test) and preference traits (the Global Preference Survey) are experimentally validated^44,45^.

The first key methodological choice of our research is the multiple measurements (in both wave one and wave two) of the exposure to shocks. We measure labor shocks as having suffered a negative change in earning or wage either the week before the first interview or the week before the current interview; stressful events shock as having suffered more than the median of the stressful events over the two weeks; health shocks as having visited a doctor, sought to be tested, or called the health service in response to COVID-19 or experienced severe stress, anxiety and depression in the previous week; *economic vulnerability predicted mental health shock* (hereafter, the label we use) as being predicted to be under severe stress, anxiety and depression conditional on economic vulnerability and negative events. The second methodological strength is the use of a randomized assignment to (placebo controlled) recall of negative emotions and stressful events. Treatment group one was asked to recall fearful or anxious events, treatment group two was asked to recall a stressful event, while the control group was asked to recall a neutral or joyful event.

On the basis of the review of the literature, we hypothesize that negative shocks hamper cognitive function, increase risk aversion and time discounting, and induce more pro-sociality than control. Our hypothesis is that stress and negative emotions drive these results: if this is the case, we expect negative emotions and stress recalls having a greater impact upon individuals exposed to shocks in comparison to those who are not exposed.

## Results

In total, 4890 subjects participated in the wave two survey, from Italy (N = 1652), Spain (N = 1660), and the UK (N = 1578) over eight days between 2–9 May 2020. For all of these subjects, we matched the data from the first wave (one week earlier) to collect the measures of socio-demographics (age, gender, education, income, employment status, residential space, household size) and the measures of shocks. These shocks included the following: a measure of behavioral change as a response to COVID-19 (sought to be tested, called a doctor, visited a doctor, called the health service), a measure of stressful events (homeschooling, did not have enough food, etc., see SOM, Sect. 2, Q17), a measure of stress, anxiety and depression (see SOM, Sect. 2, Q26), and a measure of negative labor market shock (having been fired, a reduction in earnings, closure of the company, etc., see SOM, Sect. 2, Q27). Additionally, from our companion paper on the same data^46^, we have a measure of the likelihood of being under stress, anxiety and depression, conditional on being economically vulnerable and having been exposed to a shock, using a machine learning algorithm.

For wave two, we have used the same scale of stress, anxiety and depression (SOM, Sect. 1, Q13) of a labor market shock (SOM, Sect. 1, Q14) and stressful events (SOM, Sect. 1, Q12), among the same group of participants.

We have computed the following dummies for exposure to shocks: labor shock—having suffered a reduction in earning or wage either the week before the first interview or the week before the current interview; stressful events shock—having suffered more than the median of the stressful events over the two weeks; health shock—having carried out any of the actions in response to COVID-19 or having been under severe stress, anxiety and depression in the week before; and economic vulnerability predicted mental health shock—having been predicted to be under severe stress, anxiety and depression, conditional on economic vulnerability and negative shocks.

We use the following experimental treatments. In wave two, before answering any questions, the participants were administered a recall question. Treatment group one was asked to recall a fearful or anxious episode, treatment group two was asked to recall a stressful event, and the control group was asked to recall either a neutral or a joyful event. We used both the joyful and neutral recall for two reasons: first, in case any of the shocks were particularly salient, it may be recalled as well in the neutral condition, which may harm having the correct control group (however, this turned out not to be a problem); and second, in case the statistical analysis of the recall showed causal effect, we can test for a false positive using the joy recall as a placebo experiment. These recalls are adapted from the literature on the effect of trauma and violence^15,47,48^ (the phrasing is in SOM, Sect. 1, Recalls).

After this initial question, participants were presented with three questions from the Cognitive Reflection Test and a set of experimentally validated questions to measure risk aversion, time discounting, altruism, trust, and positive and negative reciprocity. Besides risk aversion and time discounting, which we have already defined, the set of social preferences measures the following concepts: altruism—the willingness to sacrifice own payoff to the benefit of others in choices that are distributive and non-strategic; trust—the willingness to put material resources at the disposal of other persons; positive reciprocity—the willingness to face costs to repay actions that are motivated by good intentions; and negative reciprocity—the willingness to punish behavior that deviates from social norms. For the case of Altruism, we have two questions that can be used: the first asks for the share of a windfall endowment that the participant would be ready to share; the second asks for participant’s “willingness to share with others without expecting anything in return” on a Likert scale. Since we do not have reasons to prefer one or the other, we used both. For simplicity, they will be named Altruism 1 and Altruism 2, respectively.

In SOM, Sect. 3, Table S1, we report the dependent variables broken down by experimental conditions. In Column (5), we perform Pearson’s chi-squared test to assess whether or not we can treat joy recall and neutral recall together: indeed, we find that we cannot reject the null hypothesis that there is no difference. In SOM, Sect. 3, Table 2, we present descriptive statistics broken down per experimental condition and a test of balancing of the covariates (column (6), Pearson’s chi-squared test): as expected, given random allocation, there are no systematic differences across experimental conditions for any of the covariates (age, gender, education, income, employment status, residential space, household size).

**Figure 1:**
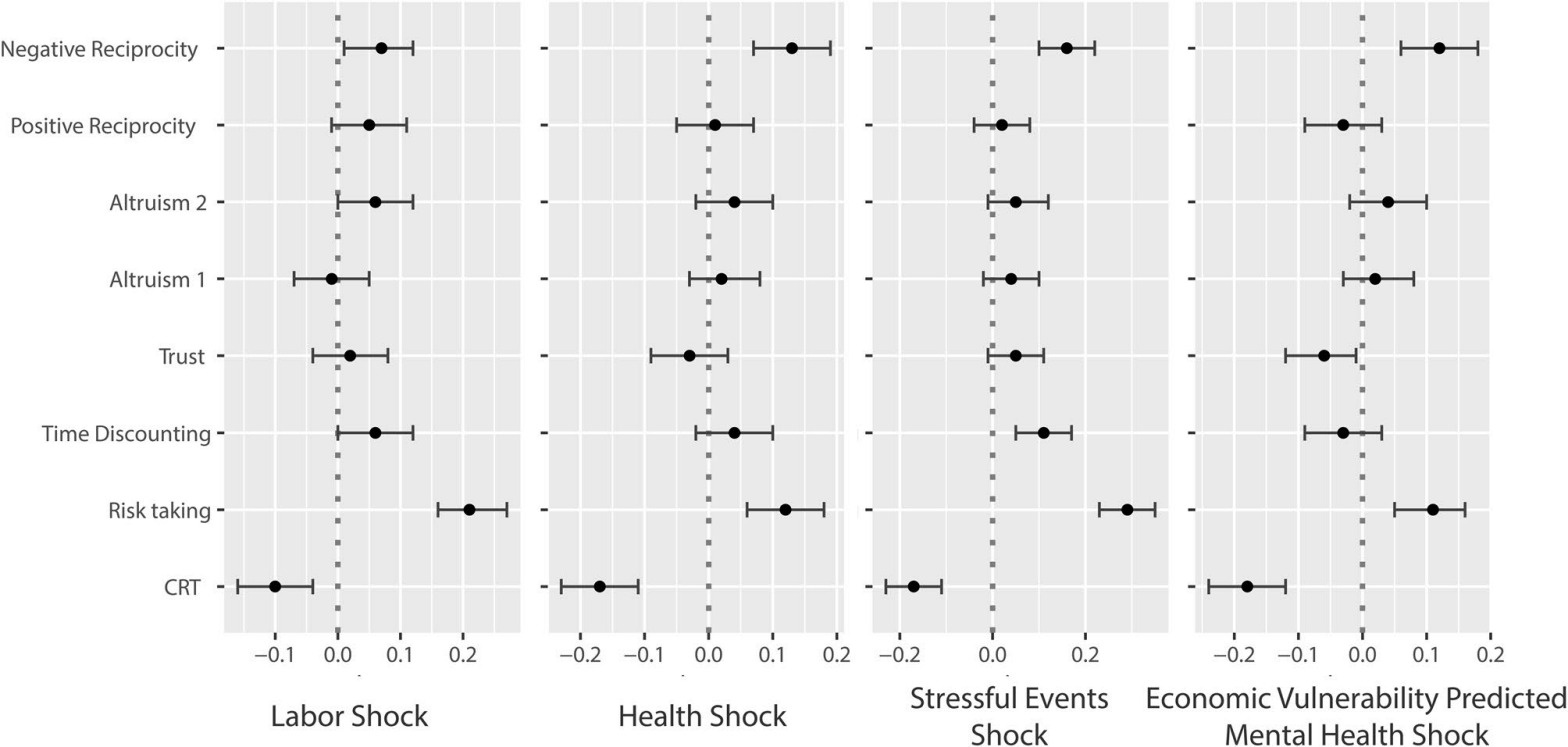
Estimated effect size for the outcome variables and the four measures of shocks (confidence interval at 95%). Note. Source: longitudinal multi-country survey conducted in Italy (N = 1,652), Spain (N = 1,660) and the United Kingdom (N = 1,578). OLS Regressions with Romano-Wolff confidence interval. Outcomes: CRT is the three items Cognitive Reflection Test. Risk Taking measures how much the participant is willing to take risks; Time discounting the willingness to give up something today in order to benefit from that in the future; Trust is the propensity to assume that people have only the best intentions; Altruism 1 is the share of a windfall endowment that the participant would be ready to share; Altruism 2 is participant’s willingness to share with others without expecting anything in return; Positive reciprocity is the choice (among six options) of a thank-you gift; Negative reciprocity is the willing to punish unfair behavior even if this is costly.

In the results that we are reporting, for the regression analysis of each dependent variable, we used the dummy for shocks as the main independent variable, we condition on the covariates and the two recalls of negative emotions and stress, and we control for Multiple Hypotheses Testing estimating Romano and Wolff p-values (RW). In Fig. 1 below, we report the estimated effect size for the outcomes separately, for each of the four measures of shocks. We systematically detect a negative effect of shocks on cognitive performance, a positive effect on risk propensity and a positive effect on negative reciprocity. All of these effects are robust to Multiple Hypotheses Testing, except for negative reciprocity with labor shock. For the cognitive performance, labor market shock reduces accuracy by − 0.1 of a standard deviation (t = − 3.20, RW = 0.013), health shock by − 0.17 of a standard deviation (t = − 5.70, RW = 0.000), stressful events shock by − 0.17 of a standard deviation (t = − 5.62, RW = 0.000), and economic vulnerability predicted mental health shock by − 0.18 of a standard deviation (t = − 6.18, RW = 0.000). For the risk propensity scale, labor market shock increases propensity by 0.21 of a standard deviation (t = 7.25, RW = 0.000), health shock by 0.12 of a standard deviation (t = 4.03, RW = 0.000), stressful events shock by 0.29 of a standard deviation (t = 9.41, RW = 0.000), and economic vulnerability predicted mental health shock by 0.11 of a standard deviation (t = 3.68, RW = 0.002). For negative reciprocity, labor market shock increases the propensity to punish a deviant behavior by 0.07 of a standard deviation (t = 2.16, RW = 0.164), health shock by 0.13 of a standard deviation (t = 4.32, RW = 0.000), stressful events shock by 0.16 of a standard deviation (t = 5.09, RW = 0.000), and economic vulnerability predicted mental health shock by 0.12 of a standard deviation (t = 3.88, RW = 0.002). Supporting regressions are reported in SOM, Sect. 3, Tables S3-S6. Stressful events’ shocks show an additional positive effect on patience (t = 3.66, RW = 0.004). However, neither health shock (t = 1.23, RW = 0.203), economic vulnerability predicted mental health shock (t = -0.98, RW = 0.701) nor labor shock (t = 2.02, RW = 0.203) show the same result.

To assess the hypothesis that stress and negative emotions are the causal mechanism behind these differences in cognitive performance and preferences after being exposed to a shock, we analyze the effect of the experimental manipulation. We performed difference-in-difference regressions: outcome variables are regressed over a dummy for shock, two dummies for negative emotions recall and stress recall, and a dummy for the interaction between shock and the negative emotions recall or the stress recall. All of the regressions include the standard covariates (age, gender, education, income, employment status, residential space, household size). The coefficient of interest is that of the interaction term: this should be interpreted as the difference in outcome between those recalling negative events and those recalling joyful or neutral events under shocks, with respect to the difference in outcome between those recalling negative events and those recalling joyful or neutral events without having been exposed to shock (i.e. the expected value of $\left[\left({Y}_{i}^{tr,1}-{Y}_{i}^{ctrl,1}\right)-\left({Y}_{i}^{tr,0}-{Y}_{i}^{ctrl,0}\right)|{X}_{i}\right],$ where *i* is the subject, *Y* the outcome variable, *tr* and *ctrl* refers to the experimental conditions, 0 and 1 to the shock status, and *X* is our set of covariates). Data fail to reject the null hypothesis that there is no negative affect or stress effect.

We report the results for the outcomes that differ according to exposure to shock: cognitive performance, risk aversion and negative reciprocity for all measures of shocks, and time discounting for stressful events’ shocks. For cognitive performance, the null hypothesis of the absence of effect of the recall in those suffering shocks compared with those who did not, is not rejected when we use labor market shock (t = 1.02, RW = 0.904), health shock (t = − 1.06, RW = 0.897), stressful events shock (t = 0.63, RW = 0.91), and economic vulnerability predicted mental health shock (t = − 1.51, RW = 0.589). For risk propensity, we similarly cannot reject the null hypothesis when we use labor market shock (t = − 0.98, RW = 0.904), health shock (t = 0.35, RW = 0.998), stressful events shock (t = − 0.85, RW = 0.91), and economic vulnerability predicted mental health shock (t = 1.31, RW = 0.695). Finally, for negative reciprocity, we confirm the same finding when we use labor market shock (t = − 1.24, RW = 0.839), health shock (t = 0.01, RW = 0.998), stressful events shock (t = − 1.78, RW = 0.397), and economic vulnerability predicted mental health shock (t = 0.39, RW = 0.902). For the time discounting in case of stressful events shock, we cannot reject the null hypothesis (t = − 1.03, RW = 0.886).

For the other outcome variables, regardless of the way we measure shocks, there is no significant interaction term. Supporting regressions are in SOM, Sect. 3, Tables S7-S10. In SOM, Tables S11-S14, we also report the regressions with the double interactions (shock times negative affect recall and shock times stress recall), where results do not change.

In Table 1 below, we summarize these findings.

**Table 1:** Coefficients for the interaction terms for each of the shock variables per dependent variable.

	Variable	CRT	Risk Taking	Time Discounting	Trust	Altruism 1	Altruism 2	Positive Reciprocity	Negative Reciprocity
(1)	Shock # Recall	0.06	− 0.15	− 0.02	0.03	0	− 0.03	0.04	− 0.16
		(0.06)	(0.15)	(0.11)	(0.14)	(0.01)	(0.14)	(0.1)	(0.13)
	Labor Shock	− 0.13***	0.66***	0.13	0.03	0	0.16	0.06	0.25**
		(0.05)	(0.13)	(0.09)	(0.12)	(0.01)	(0.12)	(0.08)	(0.11)
	Recall (Neg. Emotions)	− 0.06	− 0.01	0.06	− 0.14	0	0	0	0.13
		(0.05)	(0.13)	(0.09)	(0.12)	(0.01)	(0.12)	(0.08)	(0.11)
	Recall (Stress)	− 0.08	0.01	0.03	− 0.18	0	− 0.1	0	0.19*
		(0.05)	(0.13)	(0.09)	(0.12)	(0.01)	(0.12)	(0.08)	(0.11)
(2)	Shock # Recall	− 0.06	0.05	− 0.21*	0	0	− 0.09	− 0.03	0
		(0.06)	(0.15)	(0.11)	(0.14)	(0.01)	(0.14)	(0.1)	(0.13)
	Health Shock	− 0.12**	0.27**	0.21**	− 0.07	0.01	0.15	0.03	0.28***
		(0.05)	(0.13)	(0.09)	(0.12)	(0.01)	(0.12)	(0.08)	(0.11)
	Recall (Neg. Emotions)	0.01	− 0.13	0.18*	− 0.13	0	0.03	0.04	0.04
		(0.05)	(0.13)	(0.09)	(0.11)	(0.01)	(0.12)	(0.09)	(0.11)
	Recall (Stress)	− 0.01	− 0.11	0.15	− 0.17	0.01	− 0.07	0.05	0.1
		(0.05)	(0.13)	(0.09)	(0.11)	(0.01)	(0.12)	(0.08)	(0.1)
(3)	Shock # Recall	0.04	− 0.13	− 0.12	− 0.09	− 0.01	− 0.30**	− 0.08	− 0.23*
		(0.06)	(0.15)	(0.11)	(0.14)	(0.01)	(0.14)	(0.1)	(0.13)
	Stressful Events Shock	− 0.19***	0.83***	0.29***	0.18	0.01	0.33***	0.09	0.49***
		(0.05)	(0.13)	(0.09)	(0.12)	(0.01)	(0.12)	(0.09)	(0.11)
	Recall (Neg. Emotions)	− 0.04	− 0.03	0.12	− 0.07	0	0.16	0.07	0.17
		(0.05)	(0.13)	(0.09)	(0.12)	(0.01)	(0.12)	(0.08)	(0.11)
	Recall (Stress)	− 0.06	0	0.09	− 0.11	0.01	0.06	0.08	0.24**
		(0.05)	(0.13)	(0.09)	(0.12)	(0.01)	(0.12)	(0.08)	(0.1)
(4)	Shock # Recall	− 0.09	0.2	− 0.11	0.24*	0	0.08	− 0.08	0.05
		(0.06)	(0.15)	(0.11)	(0.14)	(0.01)	(0.14)	(0.1)	(0.13)
	Economic Vulnerability Predicted Mental Health Shock	− 0.12**	0.15	0.02	− 0.31***	0	0.03	0.01	0.22**
		(0.05)	(0.12)	(0.09)	(0.12)	(0.01)	(0.11)	(0.08)	(0.11)
	Recall (Neg. Emotions)	0.02	− 0.19*	0.1	− 0.26**	0	− 0.06	0.06	0.02
		(0.05)	(0.12)	(0.09)	(0.1)	(0.01)	(0.11)	(0.08)	(0.1)
	Recall (Stress)	0	− 0.17	0.07	− 0.29***	0	− 0.16	0.06	0.08
		(0.05)	(0.12)	(0.08)	(0.1)	(0.01)	(0.11)	(0.08)	(0.1)

Each of the cells correspond to the coefficients and their standard errors in parenthesis from regressions, including an interaction term between shock and recalls, a dummy for shock and dummies for each of the recalls. (1)-(4) corresponds to different regressions, each one with the full set of covariates. Source: longitudinal multi-country survey conducted in Italy (N = 1,652), Spain (N = 1,660) and the United Kingdom (N = 1,578). OLS Regressions with Romano-Wolff confidence interval. Outcomes: CRT is the three items Cognitive Reflection Test. Risk Taking measures how much the participant is willing to take risks; Time discounting the willingness to give up something today in order to benefit from that in the future; Trust is the propensity to assume that people have only the best intentions; Altruism 1 is the share of a windfall endowment that the participant would be ready to share; Altruism 2 is participant’s willingness to share with others without expecting anything in return; Positive reciprocity is the choice (among six options) of a thank-you gift; Negative reciprocity is the willing to punish unfair behavior even if this is costly.

**Figure 2:**
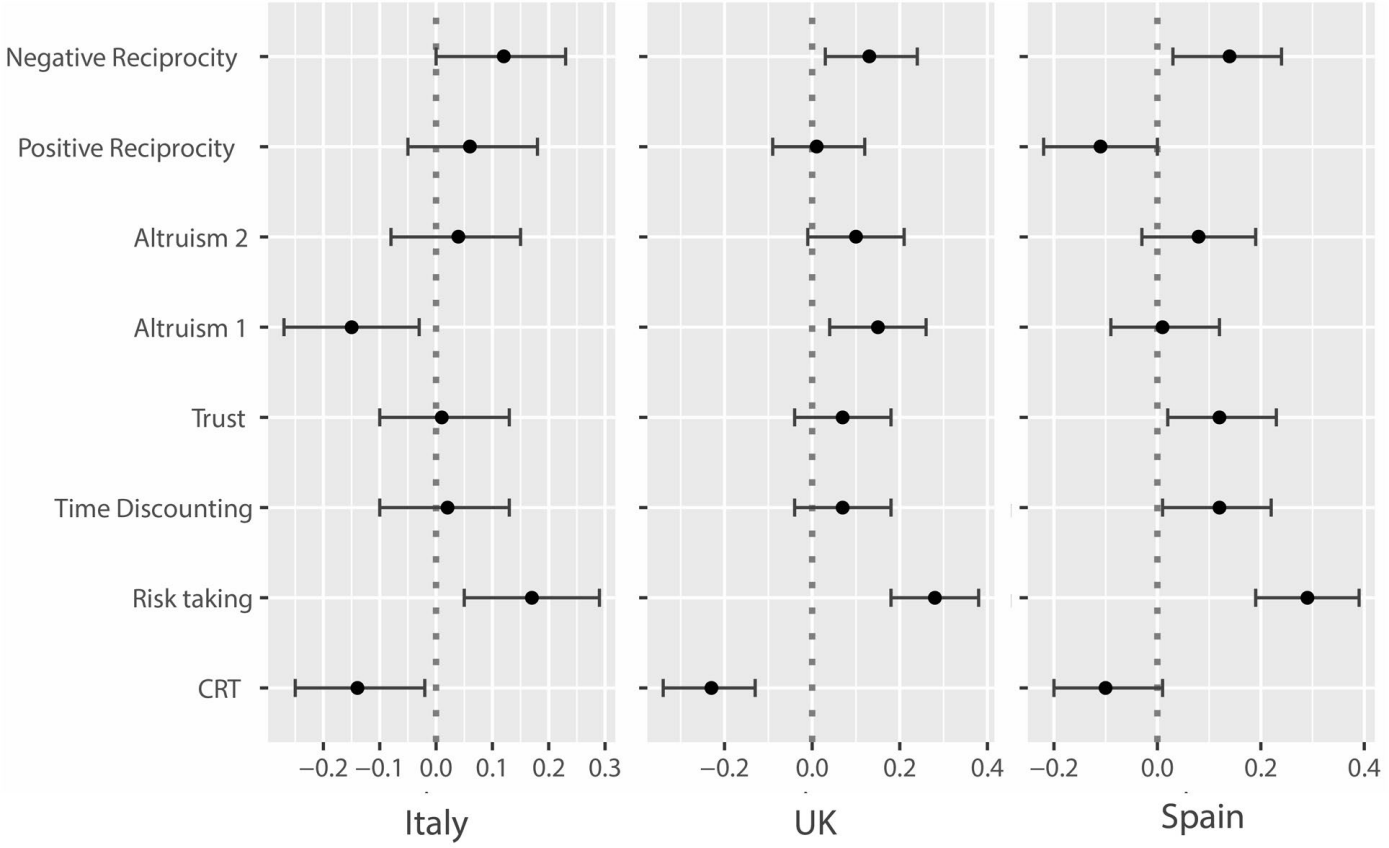
Estimated effect size of stressful events on the outcome variables by country (confidence interval at 95%). Note. Source: longitudinal multi-country survey conducted in Italy (N = 1,652), Spain (N = 1,660) and the United Kingdom (N = 1,578). OLS Regressions with Romano-Wolff confidence interval. Outcomes: CRT is the three items Cognitive Reflection Test. Risk Taking measures how much the participant is willing to take risks; Time discounting the willingness to give up something today in order to benefit from that in the future; Trust is the propensity to assume that people have only the best intentions; Altruism 1 is the share of a windfall endowment that the participant would be ready to share; Altruism 2 is participant’s willingness to share with others without expecting anything in return; Positive reciprocity is the choice (among six options) of a thank-you gift; Negative reciprocity is the willing to punish unfair behavior even if this is costly.

Finally, we analyze whether there are differences between the three countries, and indeed we find some heterogeneity. In Fig. 2 below, we report the effect size per country in percentage of standard deviation, using stressful events as a measurement of shock. In Italy, shock reduces cognitive performance by − 0.14 of a standard deviation (t = − 2.34, RW = 0.079), increases risk propensity by 0.17 of a standard deviation (t = 2.87, RW = 0.019) and decreases altruism by − 0.15 of a standard deviation (t = − 2.42, RW = 0.077). In Spain, participants affected by shock have a higher risk propensity by 0.29 of a standard deviation (t = 5.56, RW = 0.000) and more negative reciprocity by 0.14 of a standard deviation (t = 2.57, RW = 0.058). In the UK, respondents affected by shock have a lower cognitive performance by − 0.23 of a standard deviation (t = − 4.51, RW = 0.000), a higher risk propensity by 0.28 of a standard deviation (t = 5.32, RW = 0.000), more altruism by 0.15 of a standard deviation (t = 2.72, RW = 0.032), and more negative reciprocity by 0.13 of a standard deviation (t = 2.42, RW = 0.077).

Results at the country level are less consistent across measures, especially in Italy. In fact, for example, labor shocks have qualitatively the same results, but are not statistically significant, and health shocks negatively affect cognitive functioning (t = − 2.45, RW = 0.094), whereas economic vulnerability predicted mental health shock does not correlate with changes in cognitive performance or preferences. This is less the case in Spain and in the UK. By looking at differences across countries, we should be cautious since the power is reduced for each test, since we are using just a portion of the sample. All supporting regressions are reported in SOM, Sect. 3, Tables S15-S17. In the same tables, we report the results of the difference-in-difference estimation. Again, data fail to reject the null hypothesis of no stress and negative emotions effects.

## Discussion

In this article, we report results from a longitudinal multi-country study (Italy, Spain and the UK). We randomized participants to recall fearful or stressful events and we measured exposure to labor market, health, or other types of shocks. As outcome variables, we measured cognitive function using the Cognitive Reflection Test and preferences traits using an experimentally validated set of questions on risk, time, and social preferences.

Regardless of how it was measured, we found that a negative shock predicted lower cognitive function, more risk aversion, and more propensity to punish (negative reciprocity). When we assessed the negative affect and stress hypothesis, we could not reject the null hypothesis of lack of an effect. Regarding the initial hypotheses, we confirmed the negative impact of shocks on cognitive performance and the positive impact on negative reciprocity, but we found the opposite result than expected for risk propensity. One possibility is that the present context of the strong economic crisis is interpreted in terms of coping with risk in order to avoid losses, and this would be consistent with increasing loss aversion. However, it is worth noting that at least two studies have had a similar result as ours: a study of violence shock in Burundi finds an increase in risk propensity^23^, and another study in India shows that betting increases in families according to the number of shocks that they have experienced (intensive margin)^49^. It is also possible that measures of risk aversion are domain specific and do not overlap across decision contexts, in this case, the effect should be interpreted as a specific form of risk propensity^50,51^. The findings reject the initial hypothesis that the effect of shocks on outcome variables is induced by negative emotions and stress. We have focused on this explanation because it parsimoniously predicts the effect of negative shocks on cognitive, time and risk preferences and social preferences. It is possible that the recall manipulation is too mild to generate an impact (there are obvious limitations in terms of what can be done within the ethical limits of experiments and the logistical limits of online surveys), so we cannot exclude that with stronger manipulations, the null hypothesis can be rejected.

As reviewed in the Introduction, there is quasi-experimental evidence that shows an increase in anti-social behavior (petty crimes and property crimes) after negative shocks^29–32^, but those results do not contradict our results for two reasons: first, in the anti-social behavior literature, the results are mediated by weak institutions; second, an increase in anti-social behavior under shock is compatible with any set of preferences, simply if the opportunity costs of misbehaving for some individuals change dramatically.

This study has implications for the literature on the study of shocks because it provides evidence with plausible external validity, which complements the results of experiments where shocks are randomly administered but in a less externally valid setting^19^. If we assume that preferences have an idiosyncratic component which is subject to shocks, we show what is the direction of the effect of those shocks, and this has important implications for the literature on endogenous preferences^52^, the classic claim of which is that institutional settings have long-term implications for the evolution of values and tastes. Of course, we are not able to claim yet that this change is long term, but this is certainly an important question for future studies. Exposure to shock is heterogeneous both in intensity and diffusions but is reasonably common: the fact that cognitive performance and preferences are shaped by this experience may have important consequences for how we model the evolution of our societies. Finally, it has important implications in terms of public health: the current pandemic and the mitigation strategies (lockdowns) have produced significant consequences in terms of labor market and health shocks, and it is important to provide creative policy solutions for the future and to anticipate these changes, in the design of a future response to pandemics. An important strength of this study is that we were able to separately analyze the effect of inducing stress or negative emotions, the effect of having suffered a shock, and the combination of the two. By providing an exogenous source of variation through experimental conditions, this design controls for confounding factors and is better suited to an analysis of causal impact.

As usual, this study has some limitations. Ideally, one should measure cognitive performance and elicit preferences using induced value theory^53,54^. Without properly incentivized tasks, responses can be noisier, and this may reduce the statistical power^55^. Nevertheless, the use of experimentally validated questions is a strength of this study and suggests strong construct validity; also, at least for CRT, monetary incentives do not impact performance^56^. Notice also that CRT carries over the boundaries of a measurement of cognitive abilities and thinking dispositions^57^. Nevertheless, it correlates with many other measures of cognitive performance^44^, and predict performance in many heuristics-and-biases tasks^57^.

The three countries under examination (Italy, Spain and the UK) are considered to be countries that have mismanaged the overall pandemic; thus, one may wonder to what extent these results are generalizable to countries where the shock has been dealt with more efficiently. However, Italy, Spain, and the UK are also the countries in Europe where the pandemic and the lockdown have had the strongest impacts, and this suggests that confounding factors are less salient in driving the results of the effect of shocks. Current findings therefore contribute significantly to the existing literature that examines how people respond to difficult and emerging times, whereby the current COVID-19 situation is an extraordinarily strong example.

## Materials and methods

We submit a link to a random sample of participants to an online panel in three countries: Italy, Spain, and the UK. The study is preregistered on OSF: https://bit.ly/2WjzOUe. The English version of the questionnaire can be found in the Supplementary Online Materials, Sect. 1. Ethics approval was obtained from the Institutional Review Board of the Universitat Oberta de Catalunya. All respondents provided informed consent. All methods were performed in accordance with the relevant guidelines and regulations by Nature Scientific Reports.

All participants answered two online questionnaires over the course of two weeks. The wave one questionnaire included socio-demographics, self-reported health status, labor market status, and exposure to shock (SOM, Sect. 2 Questions 1–13, 18–21, 27, 24–26). Wave one is described and analyzed in Codagnone et al^46^. All questions are standardized and psychometrically validated.

Those participants who agreed to take part in wave two were randomly assigned to four conditions: Recall of Negative Emotions, Recall of Stress, Recall of Positive Emotion, Neutral Recall (SOM, Sect. 1, Recalls). The phrasing is taken from Callen et al^48^ and Bogliacino et al^15^, and adjusted with COVID-19 and lockdown related examples. The assignment criterion is the following: 30% is assigned to Recall of Negative Emotions, 30% to Recall of Stress, 20% to Recall of Positive Emotions, and 20% to Neutral Recall. The recall is shown just before answering the outcome variable questions.

Outcome variables are cognitive performance and preference traits. Cognitive performance is measured through a three-questions version of the Cognitive Reflection Test^44^ (SOM, Sect. 1, Questions 1–3). Risk and time preferences, altruism, trust, positive and negative reciprocity are measured through the Falk et al^45^ Global Preference Survey (SOM, Sect. 1, Questions 4–10).

Exposure to shock is measured in the following way: labor market shock—having suffered a change in wage or earnings over the previous two weeks; stressful events shock—having suffered more than the median of the stressful events over the previous two weeks; health shock—having carried out any of the actions in response to COVID-19 or having been under severe stress, anxiety and depression the week before; and economic vulnerability predicted mental health shock—being predicted to be under severe stress, anxiety and depression conditional on economic vulnerability and negative shocks. To estimate the relationship between the factors of economic vulnerability and exposure to negative economic shocks and stress, anxiety and depression, a random forest model is used, with bootstrapping (550 iterations). The following are the predictors used in the model: household income, a dummy for unemployed, a dummy for homeownership, living space, household size, number of children of school age, financial buffer stock, negative events that occurred in the previous week, and change in income or earnings (SOM, Sect. 2, Wave 1 Questionnaire, Q5, Q7, Q9, Q10, Q11, Q13, Q17a/b/c/d/e/f/h/i, Q27)^46^.

We analyze data using Ordinary Least Squares (OLS) regressions with robust standard errors, with the following covariates: age, sex, education, income, employment status, residence space, household size. We control for Multiple Hypotheses Testing using Romano and Wolff standard errors^58^.

## Supplementary Information

Supplementary Information.
