# Supplementary material for: Negative shocks predict change in cognitive function and preferences: assessing the negative affect and stress hypothesis
Source: Sci Rep. 2021 Feb 11;11:3546. doi: 10.1038/s41598-021-83089-0 (PMC7878761; doi:10.1038/s41598-021-83089-0)
Supplement: Supplementary file 1 — Supplementary Information. [file 41598_2021_83089_MOESM1_ESM.pdf]

Negative shocks predict change in cognitive function and preferences: Assessing the negative affect and stress hypothesis

Supplementary Online Materials

Francesco Bogliacino, Cristiano Codagnone, Felipe Montealegre, Frans Folkvord, Camilo Gómez, Rafael Charris, Giovanni Liva, Francisco Lupiáñez Villanueva, Giuseppe A. Veltri

Table of contents

Section 1. Wave 2 Questionnaire……………………………………………………… **20**

Section 2. Wave 1 Questionnaire (Questions used in this study)………................ **25**

Section 3. Supplementary Statistical Analysis……………………………………… **31**

Section 1. Wave 2 Questionnaire

Recalls. We would like you to carefully answer the following question.

Negative emotions recall

We are interested in understanding the everyday experiences that may make you fearful or anxious. This could be anything, for example having been affected by COVID-19 directly (i.e. being hospitalized) or indirectly (i.e. relatives friends hospitalized or passed away), etc. Can you describe one event in the past year that caused you fear or anxiety? Please use the space below to write your answer.

Stress recall

We are interested in understanding your everyday experiences in feeling stressed and worrisome. A lot of people are currently reporting negative emotions or stress because of the possible economic losses associated with the virus or the worsening of the economic situation. It can be anything, for example losing the job due to the negative consequences of COVID-19, having household income abruptly reduced because of the lockdown etc. Can you describe what causes you stress and worry when thinking about the current situation or the future? Please use the space below to write your answer.

Neutral Recall

We are interested in understanding your general daily experiences. This could be anything. Could you please describe one event in the past year? Please use the space below to write your answer.

Joy Recall

We are interested in understanding your daily experiences that make you happy or joyous. This could be anything, for example the birth of a child, marriage of a relative, or success in your studies or in your work. Could you please describe one event in the past year that caused you happiness? Please use the space below to write your answer.

Outcomes:

1) A bat and a ball cost $1.10 in total. The bat costs $1.00 more than the ball. How much does the ball cost? _____ cents.
2) If it takes 5 machines 5 minutes to make 5 widgets, how long would it take 100 machines to make 100 widgets? _____ minutes.
3) In a lake, there is a patch of lily pads. Every day, the patch doubles in size. If it takes 48 days for the patch to cover the entire lake, how long would it take for the patch to cover half of the lake? _____ days.
4) How do you see yourself: are you a person who is generally willing to take risks, or do you try to avoid taking risks? Please use a scale from 0 to 10, where a 0 means you are "completely unwilling to take risks" and a 10 means you are "very willing to take risks". You can also use the values in-between to indicate where you fall on the scale.

Slider from 0 to 10

5) In comparison to others, are you a person who is generally willing to give up something today in order to benefit from that in the future or are you not willing to do so? Please use a scale from 0 to 10, where a 0 means you are "completely unwilling to give up something today" and a 10 means you are very willing to give up something today". You can also use the values in between to indicate where you fall on the scale.

Slider from 0 to 10

6) How well does the following statement describe you as a person? As long as I am not convinced otherwise, I assume that people have only the best intentions. Please use a scale from 0 to 10, where 0 means “does not describe me at all" and a 10 means “describes me perfectly". You can also use the values in-between to indicate where you fall on the scale.

   Slider from 0 to 10

7) Imagine the following situation: you won 1,000 Euro in a lottery. Considering your current situation, how much would you donate to charity? (Values between 0 and 1000 are allowed).

   Allow for numeric response between 0 and 1000

8) How do you assess your willingness to share with others without expecting anything in return when it comes to charity? Please use a scale from 0 to 10, where 0 means you are “completely unwilling to share" and a 10 means you are “very willing to share". You can also use the values in between to indicate where you fall on the scale.

   Slider from 0 to 10

9) Imagine the following situation: you are shopping in an unfamiliar city and realize you lost your way. You ask a stranger for directions. The stranger offers to take you with their car to your destination. The ride takes about 20 minutes and costs the stranger about 20 Euro in total. The stranger does not want money for it. You carry six bottles of wine with you. The cheapest bottle costs 5 Euro, the most expensive one 30 Euro. You decide to give one of the bottles to the stranger as a thank-you gift. Which bottle do you give? Respondents can choose from the following options: The bottle for 5, 10, 15, 20, 25, or 30 Euro
   a. The bottle for 5 euros
   b. The bottle for 10 euros
   c. The bottle for 15 euros
   d. The bottle for 20 euros
   e. The bottle for 25 euros
   f. The bottle for 30 euros

10) How do you see yourself: Are you a person who is generally willing to punish unfair behavior even if this is costly? Please use a scale from 0 to 10, where 0 means you are “not willing at all to incur costs to punish unfair behavior" and a 10 means you are “very willing to incur costs to punish unfair behavior". You can also use the values in-between to indicate where you fall on the scale.

   Slider from 0 to 10.

11) How much would the following factors prevent you from fully isolating yourself?
   a. Need to earn an income
      a) Very Unlikely
      b) Somewhat Unlikely
      c) Somewhat Likely
      d) Very Likely
   b. Need to care for others outside your home, such as elderly parents
      a) Very Unlikely
      b) Somewhat Unlikely
      c) Somewhat Likely
      d) Very Likely
   c. Don't want to miss certain social events / gatherings
      a) Very Unlikely
      b) Somewhat Unlikely
      c) Somewhat Likely

d) Very Likely

d. Urge to practice sports
   a) Very Unlikely
   b) Somewhat Unlikely
   c) Somewhat Likely
   d) Very Likely
e. Need to leave the house for some time (for family tensions, psychological stress, boredom)
   a) Very Unlikely
   b) Somewhat Unlikely
   c) Somewhat Likely
   d) Very Likely

12) Over the past week, have any of the following events happened to you?
   a. Forced to stay at home (in shelter)
      a) Yes
      b) No
   b. Lost your employment (job or livelihood)
      a) Yes
      b) No
   c. Decrease in earning or income
      a) Yes
      b) No
   d. Had to homeschool child(ren)
      a) Yes
      b) No
   e. Unable to access health care when needed
      a) Yes
      b) No
   f. Unable to get access to sufficient food
      a) Yes
      b) No
   g. Sought to get tested for COVID-19
      a) Yes
      b) No
   h. Had to fill application for unemployment subsidy or other government sponsored support
      a) Yes
      b) No
   i. Sought help or support from charities or other non-governmental organizations
      a) Yes
      b) No

13) How often have you been bothered by the following over the past seven days?
   a) Felt down, depressed, or hopeless about the future
      i) Most or all the time (5-7 days)

    - ii) Occasionally or a moderate amount of time (3-4 days)
    - iii) Some or a little of the time (1-2) days
    - iv) Rarely or none of the time (less than 1 day)
  - b) Felt little interest or pleasure in doing things
    - i) Most or all the time (5-7 days)
    - ii) Occasionally or a moderate amount of time (3-4 days)
    - iii) Some or a little of the time (1-2) days
    - iv) Rarely or none of the time (less than 1 day)
  - c) Felt nervous, anxious or on the edge
    - i) Most or all the time (5-7 days)
    - ii) Occasionally or a moderate amount of time (3-4 days)
    - iii) Some or a little of the time (1-2) days
    - iv) Rarely or none of the time (less than 1 day)
  - d) Had trouble falling or staying asleep, or sleeping too much
    - i) Most or all the time (5-7 days)
    - ii) Occasionally or a moderate amount of time (3-4 days)
    - iii) Some or a little of the time (1-2) days
    - iv) Rarely or none of the time (less than 1 day)
  - e) Felt bad about yourself — or that you are a failure or have let yourself or your family down
    - i) Most or all the time (5-7 days)
    - ii) Occasionally or a moderate amount of time (3-4 days)
    - iii) Some or a little of the time (1-2) days
    - iv) Rarely or none of the time (less than 1 day)
  - f) Had troubles concentrating on things
    - i) Most or all the time (5-7 days)
    - ii) Occasionally or a moderate amount of time (3-4 days)
    - iii) Some or a little of the time (1-2) days
    - iv) Rarely or none of the time (less than 1 day)
  - g) Had a physical reaction when thinking about the outbreak
    - i) Most or all the time (5-7 days)
    - ii) Occasionally or a moderate amount of time (3-4 days)
    - iii) Some or a little of the time (1-2) days
    - iv) Rarely or none of the time (less than 1 day)
  - h) Feeling tired or having little energy
    - i) Most or all the time (5-7 days)
    - ii) Occasionally or a moderate amount of time (3-4 days)
    - iii) Some or a little of the time (1-2) days
    - iv) Rarely or none of the time (less than 1 day)

14) How has your wage/earnings been affected after the COVID-19 outbreak?
  - a) No change, full pay
  - b) Reduced pay
  - c) My contract was terminated
  - d) My business has closed temporarily or definitely

e) Not paid by the company, government is subsidizing pay
f) Not paid by company, employee takes unpaid leave
g) Not paid, spending my savings/helped by acquaintances.

Section 2. Wave 1 Questionnaire (Questions used in this study)

1) How old are you?

   __ years old

2) What is your sex?
   a) Female
   b) Male
   c) Other
3) What is the highest level of education you have completed?
   a) Primary school or less
   b) High school
   c) Some years of university (not completed)
   d) University degree completed
   e) Post-graduate (master, PhD, other)
4) What is your marital status?
   a) Single (never married)
   b) Married or in civil union
   c) Divorced or Widowed
5) What is your household (yearly) income?
   a) 9.999 Euro or below
   b) 10.000 Euro – 29.999 Euro
   c) 30.000 Euro – 49.999 Euro
   d) 50.000 Euro – 149.999 Euro
   e) 150.000 Euro or above
6) Which of the following best describe the area of your primary residency?
   a) Urban
   b) Suburban
   c) Rural
7) Which of the following situations best describes your current labor market status?
   a) Employed
   b) In search of job
   c) Student
   d) Retired
   e) Other (no work/no search/no study, housekeeper, disabled non-working person)
8) Which of the following occupations best describes your current prevalent activity?
   a) DIRECTORS: Directors, Executives, Directors of a company
   b) BUSINESSMEN, HOLDERS OF ACTIVITIES: Entrepreneurs, small companies or holders of activities
   c) INTELLECTUAL PROFESSIONALS, SCIENTISTS: Physicians, Chemists, Statisticians, Computer Scientists, Engineers, Architects, Biologists, Veterinarians, Pharmacists, Doctors, Dentists, Specialists in management, commercial and banking sciences, Lawyers, Solicitors, Notaries, Magistrates, University Professors (ordinary and associate), Specialists in economic, sociological, psychological, artistic, political, philosophical and literary sciences, Journalists
   d) TEACHERS: Upper and lower secondary school teachers

e) TECHNICAL PROFESSIONALS: Physical and Chemical Technicians, Stock and Exchange Brokers, Commercial Agents, Representatives, Aircraft Pilots and Civil Aviation Technicians, Photographers, Nurses, Midwives, Dieticians, Hygienists, Paramedics, Insurance Agents, Experts, Designers, Computer Social Workers, Civil Construction Engineers, Web Operators, Programmers
f) EMPLOYEES: Administrative employees, secretarial staff, accountants, employees in direct contact with the public, cashiers, counter employees
g) TRADERS OR SERVICES: Wholesalers and managers of wholesale and retail sales, Office workers and similar, Hotel service providers and similar, Waiters, Gunsmiths, Sports, recreational and cultural service providers, Hairdressers, Beauticians, Traffic Police, State Police, Firemen, Private security guards
h) ARTISTS: Artisans, Plumbers, Electricians, Tilers, Installers, Mechanics, Appliance Repairers, Goldsmiths, Decorators, Tailors, Bakers, Carpenters, Butchers, Fruit and Vegetable Makers
i) SPECIALIZED WORKERS: Specialized workers
j) FARMERS: Farmers and agricultural workers, Breeders, Fishermen
k) MACHINE WORKERS AND DRIVERS: Industrial plant operators, founders, assembly line workers, general workers, drivers, carpenters
l) UNSKILLED PROFESSIONS: Bailiffs, Doormen, Warehouse Keepers, Delivery Workers, Street Vendors, Litter Bins, Street Sweepers, Launderers, Garages, Farmers, Craftsmen and similar workers
m) ARMED FORCES: Military of all orders and ranks

9) What is the type of dwelling occupied by your household?
a) Own, fully paid
b) Own, we are paying it
c) For rent, sublet or leasing
d) In usufruct
e) Other form of tenure (untitled possession, de facto occupant, collective property, etc.)

10) What is the useful living area of your home? (It is understood by useful living area, that included within the exteriors of the house, including the common spaces).
_______________ squared meters (m^2).

11) How many people usually live in your household?
a) Adult men (age 18 and above) [ ] persons
b) Adult women (age 18 and above) [ ] persons
c) Boy children (age 3 – 17) [ ] persons
d) Girl children (age 3 – 17) [ ] persons
e) Babies (boy) (age under 3) [ ] persons
f) Babies (girl) (age under 3) [ ] persons

12) Are there other persons not living in your household because they are currently working away from home?
a) Yes [ ] persons
b) No

13) How many children are of school age in your household?
a) [ ] children 3-11 y.o.

b) [ ] children 12-18 y.o.

14) What behavior(s) have you adopted in response to COVID-19 outbreak? In case it applies, please select more than one item.
   a) Hand washing
   b) Limiting exiting home.
   c) Cover coughs/sneezes
   d) Cleaning surfaces daily
   e) Staying home when sick
   f) Working from home
   g) Nothing
   h) Wearing face mask
   i) Changing / cancelling travel plans
   j) Making family / communication plans
   k) Stocking up home supplies and medicine

15) How much would the following factors prevent you from fully isolating yourself?
   a. Need to earn an income
      a) Very Unlikely
      b) Somewhat Unlikely
      c) Somewhat Likely
      d) Very Likely
   b. Need to care for others outside your home, such as elderly parents
      a) Very Unlikely
      b) Somewhat Unlikely
      c) Somewhat Likely
      d) Very Likely
   c. Don't want to miss certain social events / gatherings
      a) Very Unlikely
      b) Somewhat Unlikely
      c) Somewhat Likely
      d) Very Likely
   d. Urge to practice sports
      a) Very Unlikely
      b) Somewhat Unlikely
      c) Somewhat Likely
      d) Very Likely
   e. Need to leave the house for some time (for family tensions, psychological stress, boredom)
      a) Very Unlikely
      b) Somewhat Unlikely
      c) Somewhat Likely
      d) Very Likely

16) If you lose your job, for how long do you believe you could pay your bills?
   a) 1 month or less
   b) 2 -3 months

c) 4 -5 months
d) 6 months or more

17) Over the past week, have any of the following events happened to you?
   a. Forced to stay at home (in shelter)
      a) Yes
      b) No
   b. Lost your employment (job or livelihood)
      a) Yes
      b) No
   c. Decrease in earning or income
      a) Yes
      b) No
   d. Had to homeschool child(ren)
      a) Yes
      b) No
   e. Unable to access health care when needed
      a) Yes
      b) No
   f. Unable to get access to sufficient food
      a) Yes
      b) No
   g. Sought to get tested for COVID-19
      a) Yes
      b) No
   h. Had to fill application for unemployment subsidy or other government sponsored support
      a) Yes
      b) No
   i. Sought help or support from charities or other non-governmental organizations
      a) Yes
      b) No

18) How is your health in general?
   a) Very good
   b) Good
   c) Neither good nor bad
   d) Bad
   e) Very bad

19) Do you have any long-standing illness or health problem?
   a) Yes
   b) No

20) Are you undergoing a long-term medical treatment?
   a) Yes
   b) No

21) Do you have or have you ever had any of the following health problems?

a) Diabetes
b) An allergy
c) Asthma
d) Hypertension (high blood pressure)
e) Long-standing troubles with your muscles, bones and joints (rheumatism, arthritis)
f) Cancer
g) Cataract
h) Migraine or frequent headaches
i) Chronic bronchitis, emphysema
j) Osteoporosis
k) Stroke, cerebral hemorrhage
l) Peptic ulcer (gastric or duodenal ulcer)
m) Chronic anxiety or depression

22) Is someone close to you, currently experiencing long-term illness or disability?
a) Yes
b) No

23) Are you taking care of such a person?
a) Yes
b) No

24) As result of COVID-19 outbreak?
a. Have you visited a doctor?
    a) Yes
    b) No
b. Have you called a doctor and/or your health care center?
    a) Yes
    b) No
c. Have you contacted any phone number to reach the health authorities?
    a) Yes
    b) No

25) How many times did you visit a doctor during the last 12 months, before the COVID-19 outbreak?
a) A few times
b) Once
c) Never

26) How often have you been bothered by the following over the past seven days?
a) Felt down, depressed, or hopeless about the future
    i) Most or all the time (5-7 days)
    ii) Occasionally or a moderate amount of time (3-4 days)
    iii) Some or a little of the time (1-2) days
    iv) Rarely or none of the time (less than 1 day)
b) Felt little interest or pleasure in doing things
    i) Most or all the time (5-7 days)
    ii) Occasionally or a moderate amount of time (3-4 days)
    iii) Some or a little of the time (1-2) days

        iv) Rarely or none of the time (less than 1 day)
   c) Felt nervous, anxious or on the edge
        i) Most or all the time (5-7 days)
        ii) Occasionally or a moderate amount of time (3-4 days)
        iii) Some or a little of the time (1-2) days
        iv) Rarely or none of the time (less than 1 day)
   d) Had trouble falling or staying asleep, or sleeping too much
        i) Most or all the time (5-7 days)
        ii) Occasionally or a moderate amount of time (3-4 days)
        iii) Some or a little of the time (1-2) days
        iv) Rarely or none of the time (less than 1 day)
   e) Felt bad about yourself — or that you are a failure or have let yourself or your family down
        i) Most or all the time (5-7 days)
        ii) Occasionally or a moderate amount of time (3-4 days)
        iii) Some or a little of the time (1-2) days
        iv) Rarely or none of the time (less than 1 day)
   f) Had troubles concentrating on things
        i) Most or all the time (5-7 days)
        ii) Occasionally or a moderate amount of time (3-4 days)
        iii) Some or a little of the time (1-2) days
        iv) Rarely or none of the time (less than 1 day)
   g) Had a physical reaction when thinking about the outbreak
        i) Most or all the time (5-7 days)
        ii) Occasionally or a moderate amount of time (3-4 days)
        iii) Some or a little of the time (1-2) days
        iv) Rarely or none of the time (less than 1 day)
   h) Feeling tired or having little energy
        i) Most or all the time (5-7 days)
        ii) Occasionally or a moderate amount of time (3-4 days)
        iii) Some or a little of the time (1-2) days
        iv) Rarely or none of the time (less than 1 day)

27) How has your wage/earnings been affected after the COVID-19 outbreak?
   a) No change, full pay
   b) Reduced pay
   c) My contract was terminated
   d) My business has closed temporarily or definitely
   e) Not paid by the company, government is subsidizing pay
   f) Not paid by company, employee takes unpaid leave
   g) Not paid, spending my savings/helped by acquaintances.

Section 3. Supplementary statistical analysis

Table S 1: Outcome variables according to experimental conditions

	(1)	(2)	(3)	(4)	(5)	(6)
Variable	Neutral	Joy	Shock	Trauma	neutral = joy	t1=t2=t3=t4
CRT	0.64 (0.96)	0.69 (0.98)	0.62 (0.95)	0.65 (0.96)	3.02 (p = 0.389)	10.23 (p = 0.115)
Risk Taking	5.55 (2.56)	5.34 (2.61)	5.42 (2.58)	5.43 (2.61)	11.30 (p = 0.334)	20.45 (p = 0.430)
Time Discounting	6.97 (1.78)	6.85 (1.89)	6.93 (1.87)	6.96 (1.86)	9.06 (p = 0.526)	11.48 (p = 0.933)
Trust	6.05 (2.30)	6.05 (2.38)	5.87 (2.43)	5.90 (2.37)	3.00 (p = 0.981)	17.77 (p = 0.602)
Altruism 1	0.13 (0.19)	0.11 (0.17)	0.12 (0.19)	0.12 (0.18)	40.29 (p = 0.248)	111.25 (p = 0.555)
Altruism 2	6.84 (2.22)	6.64 (2.26)	6.61 (2.32)	6.71 (2.27)	9.05 (p = 0.527)	17.46 (p = 0.623)
Positive Reciprocity	3.83 (1.65)	3.82 (1.66)	3.83 (1.68)	3.82 (1.63)	2.15 (p = 0.828)	6.68 (p = 0.756)
Negative Reciprocity	6.25 (2.16)	6.01 (2.14)	6.24 (2.14)	6.19 (2.10)	11.74 (p = 0.303)	25.61 (p = 0.179)

Table S 2: Covariates according to experimental conditions and balancing test

	(1)	(2)	(3)	(4)	(5)	(6)
Variable	Neutral	Joy	Shock	Trauma	neutral = joy	t1=t2=t3=t4
Age	46.31 (13.25)	47.42 (13.42)	45.75 (13.07)	45.36 (13.47)	44.62 (p = 0.883)	136.21 (p = 0.077)
Sex	1.47 (0.50)	1.50 (0.50)	1.48 (0.50)	1.51 (0.50)	1.44 (p = 0.487)	7.29 (p = 0.122)
Education	3.17 (1.18)	3.17 (1.15)	3.22 (1.14)	3.20 (1.13)	2.72 (p = 0.606)	10.97 (p = 0.203)
Income	2.60 (0.94)	2.58 (0.94)	2.58 (0.91)	2.62 (0.92)	4.13 (p = 0.389)	6.20 (p = 0.625)
Employment	1.85 (1.22)	1.87 (1.24)	1.77 (1.17)	1.77 (1.18)	5.10 (p = 0.277)	10.20 (p = 0.251)
Residence space	224.42 (1008.48)	239.93 (981.99)	444.42 (5619.97)	2445.57 (67774.23)	132.06 (p = 0.964)	541.66 (p = 0.132)
Household size	2.99 (1.54)	2.92 (1.47)	2.96 (1.50)	2.96 (1.54)	16.65 (p = 0.340)	28.77 (p = 0.860)

Regressions

Table S 3: Effect of shocks on cognitive performance and preferences, using labor market shock as independent variable

VARIABLES	(1) CRT	(2) Risk Taking	(3) Time Discounting	(4) Trust	(5) Altruism 1	(6) Altruism 2	(7) Positive Reciprocity	(8) Negative Reciprocity
Labor Shock	-0.09***	0.56***	0.11**	0.04	-0.00	0.13*	0.08	0.14**
	(0.03)	(0.08)	(0.06)	(0.07)	(0.01)	(0.07)	(0.05)	(0.06)
Recall (Neg. Emotions)	-0.02	-0.10	0.05	-0.13	0.00	-0.02	0.02	0.03
	(0.03)	(0.09)	(0.06)	(0.08)	(0.01)	(0.08)	(0.06)	(0.07)
Recall (Stress)	-0.04	-0.07	0.02	-0.16**	0.00	-0.12	0.03	0.10
	(0.03)	(0.09)	(0.06)	(0.08)	(0.01)	(0.08)	(0.06)	(0.07)
Age	0.00**	-0.02***	0.00	0.02***	0.00	0.01**	0.02***	-0.01**
	(0.00)	(0.00)	(0.00)	(0.00)	(0.00)	(0.00)	(0.00)	(0.00)
Sex	0.25***	0.62***	-0.06	0.06	-0.01*	-0.21***	-0.09**	0.27***
	(0.03)	(0.07)	(0.05)	(0.07)	(0.01)	(0.07)	(0.05)	(0.06)
Education	0.09***	0.15***	0.09***	0.11***	0.01***	0.07**	0.04**	0.04
	(0.01)	(0.03)	(0.02)	(0.03)	(0.00)	(0.03)	(0.02)	(0.03)
Income	0.12***	0.15***	0.07**	0.11***	0.00	0.11***	0.08***	0.12***
	(0.02)	(0.04)	(0.03)	(0.04)	(0.00)	(0.04)	(0.03)	(0.04)
Employment	0.04***	-0.14***	-0.04	-0.05*	0.00	0.05*	0.05**	-0.06**
	(0.01)	(0.03)	(0.02)	(0.03)	(0.00)	(0.03)	(0.02)	(0.03)
Residence Space	-0.00**	0.00***	-0.00**	0.00***	-0.00***	0.00***	-0.00	-0.00***
	(0.00)	(0.00)	(0.00)	(0.00)	(0.00)	(0.00)	(0.00)	(0.00)
Household Size	-0.04***	0.11***	0.04*	0.07***	0.01***	0.06**	0.05**	0.04*
	(0.01)	(0.02)	(0.02)	(0.02)	(0.00)	(0.03)	(0.02)	(0.03)
Constant	-0.33***	4.43***	6.28***	4.27***	0.07***	5.91***	2.63***	5.48***
	(0.10)	(0.27)	(0.20)	(0.25)	(0.02)	(0.24)	(0.18)	(0.23)
Romano Wolf p-values	0.010	0.000	0.203	0.772	0.782	0.203	0.284	0.158
Observations	4,890	4,890	4,890	4,890	4,890	4,890	4,890	4,890
R-squared	0.06	0.08	0.01	0.02	0.01	0.01	0.02	0.02

Note: Robust standard errors in parentheses; *** p<0.01, ** p<0.05, * p<0.1. Romano Wolf p-values correspond to the shock variable. Regressions are done using individual-level control variables in Table S 2.

Table S 4: Effect of shocks on cognitive performance and preferences, using health market shock as independent variable

VARIABLES	(1)<br>CRT	(2)<br>Risk Taking	(3)<br>Time Discounting	(4)<br>Trust	(5)<br>Altruism 1	(6)<br>Altruism 2	(7)<br>Positive Reciprocity	(8)<br>Negative Reciprocity
Health Shock	-0.16***	0.31***	0.07	-0.07	0.00	0.09	0.01	0.28***
	(0.03)	(0.08)	(0.06)	(0.07)	(0.01)	(0.07)	(0.05)	(0.06)
Recall (Neg. Emotions)	-0.03	-0.10	0.05	-0.13	0.00	-0.02	0.02	0.04
	(0.03)	(0.09)	(0.06)	(0.08)	(0.01)	(0.08)	(0.06)	(0.07)
Recall (Stress)	-0.04	-0.07	0.02	-0.17**	0.00	-0.12	0.03	0.10
	(0.03)	(0.09)	(0.06)	(0.08)	(0.01)	(0.08)	(0.06)	(0.07)
Age	0.00	-0.03***	0.00	0.02***	0.00	0.01**	0.01***	-0.00*
	(0.00)	(0.00)	(0.00)	(0.00)	(0.00)	(0.00)	(0.00)	(0.00)
Sex	0.24***	0.63***	-0.05	0.05	-0.01	-0.20***	-0.09**	0.29***
	(0.03)	(0.07)	(0.05)	(0.07)	(0.01)	(0.07)	(0.05)	(0.06)
Education	0.10***	0.14***	0.09***	0.11***	0.01***	0.07**	0.04**	0.03
	(0.01)	(0.03)	(0.02)	(0.03)	(0.00)	(0.03)	(0.02)	(0.03)
Income	0.13***	0.11***	0.06*	0.10**	0.00	0.10**	0.07***	0.12***
	(0.02)	(0.04)	(0.03)	(0.04)	(0.00)	(0.04)	(0.03)	(0.04)
Employment	0.05***	-0.17***	-0.04*	-0.05*	0.00	0.05	0.04**	-0.07**
	(0.01)	(0.03)	(0.02)	(0.03)	(0.00)	(0.03)	(0.02)	(0.03)
Residence Space	-0.00***	0.00***	-0.00**	0.00***	-0.00***	0.00***	-0.00	-0.00***
	(0.00)	(0.00)	(0.00)	(0.00)	(0.00)	(0.00)	(0.00)	(0.00)
Household Size	-0.04***	0.12***	0.04*	0.07***	0.01***	0.06**	0.05**	0.04*
	(0.01)	(0.02)	(0.02)	(0.02)	(0.00)	(0.03)	(0.02)	(0.03)
Constant	-0.26***	4.75***	6.34***	4.40***	0.06***	5.97***	2.71***	5.34***
	(0.09)	(0.27)	(0.19)	(0.25)	(0.02)	(0.24)	(0.17)	(0.23)
Romano Wolf p-values	0.000	0.000	0.634	0.728	0.728	0.603	0.765	0.000
Observations	4,890	4,890	4,890	4,890	4,890	4,890	4,890	4,890
R-squared	0.06	0.07	0.01	0.02	0.01	0.01	0.02	0.02

Note: Robust standard errors in parentheses; *** p<0.01, ** p<0.05, * p<0.1. Romano Wolf p-values correspond to the shock variable. Regressions are done using individual-level control variables in Table S 2.

Table S 5: Effect of shocks on cognitive performance and preferences, using stressful events shock as independent variable

VARIABLES	(1)<br>CRT	(2)<br>Risk Taking	(3)<br>Time Discounting	(4)<br>Trust	(5)<br>Altruism 1	(6)<br>Altruism 2	(7)<br>Positive Reciprocity	(8)<br>Negative Reciprocity
Stressful Events Shock	-0.17***	0.75***	0.21***	0.12	0.01	0.12*	0.03	0.33***
	(0.03)	(0.08)	(0.06)	(0.07)	(0.01)	(0.07)	(0.05)	(0.07)
Recall (Neg. Emotions)	-0.02	-0.11	0.05	-0.13	0.00	-0.02	0.02	0.03
	(0.03)	(0.09)	(0.06)	(0.08)	(0.01)	(0.08)	(0.06)	(0.07)
Recall (Stress)	-0.04	-0.08	0.02	-0.16**	0.00	-0.12	0.03	0.10
	(0.03)	(0.09)	(0.06)	(0.08)	(0.01)	(0.08)	(0.06)	(0.07)
Age	0.00	-0.02***	0.00*	0.02***	0.00	0.01**	0.01***	-0.00*
	(0.00)	(0.00)	(0.00)	(0.00)	(0.00)	(0.00)	(0.00)	(0.00)
Sex	0.25***	0.62***	-0.06	0.06	-0.01*	-0.21***	-0.10**	0.27***
	(0.03)	(0.07)	(0.05)	(0.07)	(0.01)	(0.07)	(0.05)	(0.06)
Education	0.10***	0.14***	0.09***	0.11***	0.01***	0.07**	0.04**	0.03
	(0.01)	(0.03)	(0.02)	(0.03)	(0.00)	(0.03)	(0.02)	(0.03)
Income	0.12***	0.15***	0.07**	0.11***	0.00	0.10***	0.08***	0.13***
	(0.02)	(0.04)	(0.03)	(0.04)	(0.00)	(0.04)	(0.03)	(0.04)
Employment	0.04***	-0.13***	-0.03	-0.05	0.00	0.05*	0.04**	-0.05*
	(0.01)	(0.03)	(0.02)	(0.03)	(0.00)	(0.03)	(0.02)	(0.03)
Residence Space	-0.00**	0.00***	-0.00**	0.00***	-0.00***	0.00***	-0.00	-0.00***
	(0.00)	(0.00)	(0.00)	(0.00)	(0.00)	(0.00)	(0.00)	(0.00)
Household Size	-0.03***	0.08***	0.03	0.06***	0.01***	0.06**	0.05**	0.03
	(0.01)	(0.02)	(0.02)	(0.02)	(0.00)	(0.03)	(0.02)	(0.03)
Constant	-0.27***	4.32***	6.20***	4.20***	0.06***	5.94***	2.69***	5.30***
	(0.09)	(0.26)	(0.19)	(0.25)	(0.02)	(0.24)	(0.17)	(0.22)
Romano Wolf p-values	0.000	0.000	0.004	0.303	0.311	0.287	0.509	0.000
Observations	4,890	4,890	4,890	4,890	4,890	4,890	4,890	4,890
R-squared	0.06	0.09	0.01	0.02	0.01	0.01	0.02	0.02

Note: Robust standard errors in parentheses; *** p<0.01, ** p<0.05, * p<0.1. Romano Wolf p-values correspond to the shock variable. Regressions are done using individual-level control variables in Table S 2.

Table S 6: Effect of shocks on cognitive performance and preferences, using economic vulnerability predicted mental health shock as independent variable

VARIABLES	(1) CRT	(2) Risk Taking	(3) Time Discounting	(4) Trust	(5) Altruism 1	(6) Altruism 2	(7) Positive Reciprocity	(8) Negative Reciprocity
Economic Vulnerability Predicted Mental Health Shock	-0.17***	0.28***	-0.05	-0.15**	0.00	0.09	-0.04	0.25***
	(0.03)	(0.08)	(0.06)	(0.07)	(0.01)	(0.07)	(0.05)	(0.06)
Recall (Neg. Emotions)	-0.03	-0.09	0.04	-0.13	0.00	-0.02	0.02	0.04
	(0.03)	(0.09)	(0.06)	(0.08)	(0.01)	(0.08)	(0.06)	(0.07)
Recall (Stress)	-0.05	-0.07	0.01	-0.17**	0.00	-0.12	0.03	0.11
	(0.03)	(0.09)	(0.06)	(0.08)	(0.01)	(0.08)	(0.06)	(0.07)
Age	0.00	-0.03***	0.00	0.02***	0.00	0.01**	0.01***	-0.00*
	(0.00)	(0.00)	(0.00)	(0.00)	(0.00)	(0.00)	(0.00)	(0.00)
Sex	0.23***	0.63***	-0.07	0.04	-0.01	-0.20***	-0.10**	0.29***
	(0.03)	(0.07)	(0.05)	(0.07)	(0.01)	(0.07)	(0.05)	(0.06)
Education	0.09***	0.15***	0.09***	0.11***	0.01***	0.07**	0.04**	0.03
	(0.01)	(0.03)	(0.02)	(0.03)	(0.00)	(0.03)	(0.02)	(0.03)
Income	0.12***	0.11***	0.05	0.09**	0.00	0.10**	0.07**	0.13***
	(0.02)	(0.04)	(0.03)	(0.04)	(0.00)	(0.04)	(0.03)	(0.04)
Employment	0.05***	-0.17***	-0.05*	-0.05*	0.00	0.05	0.04**	-0.07***
	(0.01)	(0.03)	(0.02)	(0.03)	(0.00)	(0.03)	(0.02)	(0.03)
Residence Space	-0.00**	0.00***	-0.00**	0.00***	-0.00***	0.00***	-0.00	-0.00***
	(0.00)	(0.00)	(0.00)	(0.00)	(0.00)	(0.00)	(0.00)	(0.00)
Household Size	-0.04***	0.12***	0.04**	0.07***	0.01***	0.06**	0.05***	0.04*
	(0.01)	(0.02)	(0.02)	(0.02)	(0.00)	(0.03)	(0.02)	(0.03)
Constant	-0.24**	4.77***	6.49***	4.51***	0.06***	5.97***	2.78***	5.35***
	(0.10)	(0.27)	(0.19)	(0.25)	(0.02)	(0.24)	(0.18)	(0.23)
Romano Wolf p-values	0.000	0.002	0.701	0.143	0.701	0.582	0.701	0.002
Observations	4,890	4,890	4,890	4,890	4,890	4,890	4,890	4,890
R-squared	0.06	0.07	0.01	0.02	0.01	0.01	0.02	0.02

Note: Robust standard errors in parentheses; *** p<0.01, ** p<0.05, * p<0.1. Romano Wolf p-values correspond to the shock variable. Regressions are done using individual-level control variables in Table S 2.

Table S 7 Difference-in-difference estimation of the effect of negative emotions and stress, using labor market shock

VARIABLES	(1) CRT	(2) Risk Taking	(3) Time Discounting	(4) Trust	(5) Altruism 1	(6) Altruism 2	(7) Positive Reciprocity	(8) Negative Reciprocity
Shock # Recall	0.06	-0.15	-0.02	0.03	-0.00	-0.03	0.04	-0.16
	(0.06)	(0.15)	(0.11)	(0.14)	(0.01)	(0.14)	(0.10)	(0.13)
Labor Shock	-0.13***	0.66***	0.13	0.03	0.00	0.16	0.06	0.25**
	(0.05)	(0.13)	(0.09)	(0.12)	(0.01)	(0.12)	(0.08)	(0.11)
Recall (Neg. Emotions)	-0.06	-0.01	0.06	-0.14	0.00	-0.00	0.00	0.13
	(0.05)	(0.13)	(0.09)	(0.12)	(0.01)	(0.12)	(0.08)	(0.11)
Recall (Stress)	-0.08	0.01	0.03	-0.18	0.00	-0.10	0.00	0.19*
	(0.05)	(0.13)	(0.09)	(0.12)	(0.01)	(0.12)	(0.08)	(0.11)
Age	0.00**	-0.02***	0.00	0.02***	0.00	0.01**	0.02***	-0.01**
	(0.00)	(0.00)	(0.00)	(0.00)	(0.00)	(0.00)	(0.00)	(0.00)
Sex	0.25***	0.62***	-0.06	0.06	-0.01*	-0.21***	-0.09**	0.27***
	(0.03)	(0.07)	(0.05)	(0.07)	(0.01)	(0.07)	(0.05)	(0.06)
Education	0.09***	0.15***	0.09***	0.11***	0.01***	0.07**	0.04**	0.04
	(0.01)	(0.03)	(0.02)	(0.03)	(0.00)	(0.03)	(0.02)	(0.03)
Income	0.12***	0.15***	0.07**	0.11***	0.00	0.11***	0.08***	0.12***
	(0.02)	(0.04)	(0.03)	(0.04)	(0.00)	(0.04)	(0.03)	(0.04)
Employment	0.04***	-0.14***	-0.04	-0.05*	0.00	0.05*	0.05**	-0.06**
	(0.01)	(0.03)	(0.02)	(0.03)	(0.00)	(0.03)	(0.02)	(0.03)
Residence Space	-0.00***	0.00***	-0.00**	0.00***	-0.00***	0.00***	-0.00	-0.00***
	(0.00)	(0.00)	(0.00)	(0.00)	(0.00)	(0.00)	(0.00)	(0.00)
Household Size	-0.04***	0.11***	0.04*	0.07***	0.01***	0.06**	0.05**	0.04*
	(0.01)	(0.02)	(0.02)	(0.02)	(0.00)	(0.03)	(0.02)	(0.03)
Constant	-0.31***	4.37***	6.27***	4.28***	0.07***	5.90***	2.64***	5.42***
	(0.10)	(0.27)	(0.20)	(0.26)	(0.02)	(0.24)	(0.18)	(0.24)
Romano Wolf p-values	0.904	0.904	0.997	0.997	0.997	0.997	0.994	0.839
Observations	4,890	4,890	4,890	4,890	4,890	4,890	4,890	4,890
R-squared	0.06	0.08	0.01	0.02	0.01	0.01	0.02	0.02

Note: Robust standard errors in parentheses; *** p<0.01, ** p<0.05, * p<0.1. Romano Wolf p-values correspond to the interaction between shock variable and the recall. Regressions are done using individual-level control variables in Table S 2.

Table S 8 Difference-in-difference estimation of the effect of negative emotions and stress, using health market shock

VARIABLES	(1) CRT	(2) Risk Taking	(3) Time Discounting	(4) Trust	(5) Altruism 1	(6) Altruism 2	(7) Positive Reciprocity	(8) Negative Reciprocity
Shock # Recall	-0.06	0.05	-0.21*	0.00	-0.00	-0.09	-0.03	0.00
	(0.06)	(0.15)	(0.11)	(0.14)	(0.01)	(0.14)	(0.10)	(0.13)
Health Shock	-0.12**	0.27**	0.21**	-0.07	0.01	0.15	0.03	0.28***
	(0.05)	(0.13)	(0.09)	(0.12)	(0.01)	(0.12)	(0.08)	(0.11)
Recall (Neg. Emotions)	0.01	-0.13	0.18*	-0.13	0.00	0.03	0.04	0.04
	(0.05)	(0.13)	(0.09)	(0.11)	(0.01)	(0.12)	(0.09)	(0.11)
Recall (Stress)	-0.01	-0.11	0.15	-0.17	0.01	-0.07	0.05	0.10
	(0.05)	(0.13)	(0.09)	(0.11)	(0.01)	(0.12)	(0.08)	(0.10)
Age	0.00	-0.03***	0.00	0.02***	0.00	0.01**	0.01***	-0.00*
	(0.00)	(0.00)	(0.00)	(0.00)	(0.00)	(0.00)	(0.00)	(0.00)
Sex	0.24***	0.63***	-0.05	0.05	-0.01	-0.20***	-0.09**	0.29***
	(0.03)	(0.07)	(0.05)	(0.07)	(0.01)	(0.07)	(0.05)	(0.06)
Education	0.10***	0.14***	0.09***	0.11***	0.01***	0.07**	0.04**	0.03
	(0.01)	(0.03)	(0.02)	(0.03)	(0.00)	(0.03)	(0.02)	(0.03)
Income	0.13***	0.11***	0.06*	0.10**	0.00	0.10**	0.07***	0.12***
	(0.02)	(0.04)	(0.03)	(0.04)	(0.00)	(0.04)	(0.03)	(0.04)
Employment	0.05***	-0.17***	-0.05*	-0.05*	0.00	0.05	0.04**	-0.07**
	(0.01)	(0.03)	(0.02)	(0.03)	(0.00)	(0.03)	(0.02)	(0.03)
Residence Space	-0.00**	0.00***	-0.00**	0.00***	-0.00***	0.00***	-0.00	-0.00***
	(0.00)	(0.00)	(0.00)	(0.00)	(0.00)	(0.00)	(0.00)	(0.00)
Household Size	-0.04***	0.12***	0.04*	0.07***	0.01***	0.06**	0.05**	0.04*
	(0.01)	(0.02)	(0.02)	(0.02)	(0.00)	(0.03)	(0.02)	(0.03)
Constant	-0.29***	4.78***	6.26***	4.40***	0.06***	5.94***	2.70***	5.34***
	(0.10)	(0.27)	(0.20)	(0.25)	(0.02)	(0.25)	(0.18)	(0.23)
Romano Wolf p-values	0.897	0.998	0.387	0.998	0.998	0.985	0.998	0.998
Observations	4,890	4,890	4,890	4,890	4,890	4,890	4,890	4,890
R-squared	0.06	0.07	0.01	0.02	0.01	0.01	0.02	0.02

Note: Robust standard errors in parentheses; *** p<0.01, ** p<0.05, * p<0.1. Romano Wolf p-values correspond to the interaction between shock variable and the recall. Regressions are done using individual-level control variables in Table S 2.

Table S 9 Difference-in-difference estimation of the effect of negative emotions and stress, using stressful events shock

VARIABLES	(1) CRT	(2) Risk Taking	(3) Time Discounting	(4) Trust	(5) Altruism 1	(6) Altruism 2	(7) Positive Reciprocity	(8) Negative Reciprocity
Shock # Recall	0.04	-0.13	-0.12	-0.09	-0.01	-0.30**	-0.08	-0.23*
	(0.06)	(0.15)	(0.11)	(0.14)	(0.01)	(0.14)	(0.10)	(0.13)
Stressful Events Shock	-0.19***	0.83***	0.29***	0.18	0.01	0.33***	0.09	0.49***
	(0.05)	(0.13)	(0.09)	(0.12)	(0.01)	(0.12)	(0.09)	(0.11)
Recall (Neg. Emotions)	-0.04	-0.03	0.12	-0.07	0.00	0.16	0.07	0.17
	(0.05)	(0.13)	(0.09)	(0.12)	(0.01)	(0.12)	(0.08)	(0.11)
Recall (Stress)	-0.06	-0.00	0.09	-0.11	0.01	0.06	0.08	0.24**
	(0.05)	(0.13)	(0.09)	(0.12)	(0.01)	(0.12)	(0.08)	(0.10)
Age	0.00	-0.02***	0.00*	0.02***	0.00	0.01**	0.01***	-0.00*
	(0.00)	(0.00)	(0.00)	(0.00)	(0.00)	(0.00)	(0.00)	(0.00)
Sex	0.25***	0.62***	-0.06	0.06	-0.01*	-0.21***	-0.10**	0.27***
	(0.03)	(0.07)	(0.05)	(0.07)	(0.01)	(0.07)	(0.05)	(0.06)
Education	0.10***	0.14***	0.09***	0.11***	0.01***	0.07**	0.04**	0.03
	(0.01)	(0.03)	(0.02)	(0.03)	(0.00)	(0.03)	(0.02)	(0.03)
Income	0.12***	0.15***	0.07**	0.11***	0.00	0.11***	0.08***	0.13***
	(0.02)	(0.04)	(0.03)	(0.04)	(0.00)	(0.04)	(0.03)	(0.04)
Employment	0.04***	-0.12***	-0.03	-0.05	0.00	0.05*	0.04**	-0.05*
	(0.01)	(0.03)	(0.02)	(0.03)	(0.00)	(0.03)	(0.02)	(0.03)
Residence Space	-0.00**	0.00***	-0.00**	0.00***	-0.00***	0.00***	-0.00	-0.00***
	(0.00)	(0.00)	(0.00)	(0.00)	(0.00)	(0.00)	(0.00)	(0.00)
Household Size	-0.03***	0.08***	0.03	0.06***	0.01***	0.05**	0.05**	0.03
	(0.01)	(0.02)	(0.02)	(0.02)	(0.00)	(0.03)	(0.02)	(0.03)
Constant	-0.26***	4.27***	6.15***	4.17***	0.06***	5.82***	2.66***	5.21***
	(0.10)	(0.27)	(0.20)	(0.26)	(0.02)	(0.24)	(0.18)	(0.23)
Romano Wolf p-values	0.910	0.910	0.886	0.910	0.910	0.197	0.910	0.397
Observations	4,890	4,890	4,890	4,890	4,890	4,890	4,890	4,890
R-squared	0.06	0.09	0.01	0.02	0.01	0.01	0.02	0.02

Note: Robust standard errors in parentheses; *** p<0.01, ** p<0.05, * p<0.1. Romano Wolf p-values correspond to the interaction between shock variable and the recall. Regressions are done using individual-level control variables in Table S 2.

Table S 10 Difference-in-difference estimation of the effect of negative emotions and stress, using economic vulnerability predicted mental health shock

VARIABLES	(1) CRT	(2) Risk Taking	(3) Time Discounting	(4) Trust	(5) Altruism 1	(6) Altruism 2	(7) Positive Reciprocity	(8) Negative Reciprocity
Shock # Recall	-0.09	0.20	-0.11	0.24*	0.00	0.08	-0.08	0.05
	(0.06)	(0.15)	(0.11)	(0.14)	(0.01)	(0.14)	(0.10)	(0.13)
Economic Vulnerability Predicted Mental Health Shock	-0.12**	0.15	0.02	-0.31***	0.00	0.03	0.01	0.22**
	(0.05)	(0.12)	(0.09)	(0.12)	(0.01)	(0.11)	(0.08)	(0.11)
Recall (Neg. Emotions)	0.02	-0.19*	0.10	-0.26**	-0.00	-0.06	0.06	0.02
	(0.05)	(0.12)	(0.09)	(0.10)	(0.01)	(0.11)	(0.08)	(0.10)
Recall (Stress)	-0.00	-0.17	0.07	-0.29***	0.00	-0.16	0.06	0.08
	(0.05)	(0.12)	(0.08)	(0.10)	(0.01)	(0.11)	(0.08)	(0.10)
Age	0.00	-0.03***	0.00	0.02***	0.00	0.01**	0.01***	-0.00*
	(0.00)	(0.00)	(0.00)	(0.00)	(0.00)	(0.00)	(0.00)	(0.00)
Sex	0.23***	0.63***	-0.07	0.04	-0.01	-0.20***	-0.10**	0.29***
	(0.03)	(0.07)	(0.05)	(0.07)	(0.01)	(0.07)	(0.05)	(0.06)
Education	0.09***	0.15***	0.09***	0.11***	0.01***	0.07**	0.04**	0.03
	(0.01)	(0.03)	(0.02)	(0.03)	(0.00)	(0.03)	(0.02)	(0.03)
Income	0.12***	0.11***	0.05	0.10**	0.00	0.10**	0.07**	0.13***
	(0.02)	(0.04)	(0.03)	(0.04)	(0.00)	(0.04)	(0.03)	(0.04)
Employment	0.05***	-0.17***	-0.05*	-0.05*	0.00	0.05	0.04*	-0.07**
	(0.01)	(0.03)	(0.02)	(0.03)	(0.00)	(0.03)	(0.02)	(0.03)
Residence Space	-0.00**	0.00***	-0.00**	0.00***	-0.00***	0.00***	-0.00	-0.00***
	(0.00)	(0.00)	(0.00)	(0.00)	(0.00)	(0.00)	(0.00)	(0.00)
Household Size	-0.04***	0.12***	0.04**	0.07***	0.01***	0.06**	0.05***	0.04*
	(0.01)	(0.02)	(0.02)	(0.02)	(0.00)	(0.03)	(0.02)	(0.03)
Constant	-0.27***	4.83***	6.45***	4.59***	0.06***	6.00***	2.75***	5.37***
	(0.10)	(0.27)	(0.20)	(0.25)	(0.02)	(0.25)	(0.18)	(0.23)
Romano Wolf p-values	0.589	0.695	0.847	0.501	0.902	0.890	0.886	0.902
Observations	4,890	4,890	4,890	4,890	4,890	4,890	4,890	4,890
R-squared	0.06	0.07	0.01	0.02	0.01	0.01	0.02	0.02

Note: Robust standard errors in parentheses; *** p<0.01, ** p<0.05, * p<0.1. Romano Wolf p-values correspond to the coefficient of the interaction between shock variable and the recall. Regressions are done using individual-level control variables in Table S 2.

Table S 11 Difference-in-difference estimation of the effect of negative emotions and stress, using double interaction terms and labor market shock

VARIABLES	(1)<br>CRT	(2)<br>Risk Taking	(3)<br>Time Discounting	(4)<br>Trust	(5)<br>Altruism 1	(6)<br>Altruism 2	(7)<br>Positive Reciprocity	(8)<br>Negative Reciprocity
Shock # Recall (Stress)	0.05	-0.18	0.01	0.09	0.01	0.23	0.10	-0.14
	(0.07)	(0.18)	(0.13)	(0.17)	(0.01)	(0.16)	(0.12)	(0.15)
Shock # Recall (Neg. Emotions)	0.07	-0.12	-0.06	-0.04	-0.01	-0.31*	-0.03	-0.18
	(0.07)	(0.18)	(0.13)	(0.17)	(0.01)	(0.16)	(0.12)	(0.15)
Labor Shock	-0.13***	0.66***	0.13	0.03	-0.00	0.15	0.06	0.25**
	(0.05)	(0.13)	(0.09)	(0.12)	(0.01)	(0.12)	(0.08)	(0.11)
Recall (Neg. Emotions)	-0.07	-0.03	0.08	-0.10	0.01	0.16	0.04	0.14
	(0.05)	(0.14)	(0.10)	(0.13)	(0.01)	(0.13)	(0.09)	(0.12)
Recall (Stress)	-0.07	0.03	0.01	-0.22*	-0.00	-0.26**	-0.03	0.18
	(0.05)	(0.14)	(0.10)	(0.13)	(0.01)	(0.13)	(0.09)	(0.11)
Age	0.00**	-0.02***	0.00	0.02***	0.00	0.01**	0.02***	-0.01**
	(0.00)	(0.00)	(0.00)	(0.00)	(0.00)	(0.00)	(0.00)	(0.00)
Sex	0.25***	0.62***	-0.06	0.06	-0.01*	-0.21***	-0.09**	0.27***
	(0.03)	(0.07)	(0.05)	(0.07)	(0.01)	(0.07)	(0.05)	(0.06)
Education	0.09***	0.15***	0.09***	0.11***	0.01***	0.07**	0.04**	0.04
	(0.01)	(0.03)	(0.02)	(0.03)	(0.00)	(0.03)	(0.02)	(0.03)
Income	0.12***	0.15***	0.06*	0.11***	0.00	0.10***	0.08***	0.12***
	(0.02)	(0.04)	(0.03)	(0.04)	(0.00)	(0.04)	(0.03)	(0.04)
Employment	0.04***	-0.14***	-0.04	-0.05*	0.00	0.05*	0.04**	-0.06**
	(0.01)	(0.03)	(0.02)	(0.03)	(0.00)	(0.03)	(0.02)	(0.03)
Residence Space	-0.00***	0.00***	-0.00**	0.00***	-0.00**	0.00***	-0.00	-0.00***
	(0.00)	(0.00)	(0.00)	(0.00)	(0.00)	(0.00)	(0.00)	(0.00)
Household Size	-0.04***	0.11***	0.04*	0.07***	0.01***	0.06**	0.05**	0.04*
	(0.01)	(0.02)	(0.02)	(0.02)	(0.00)	(0.03)	(0.02)	(0.03)
Constant	-0.31***	4.37***	6.28***	4.28***	0.07***	5.91***	2.64***	5.42***
	(0.10)	(0.27)	(0.20)	(0.26)	(0.02)	(0.24)	(0.18)	(0.24)
Romano Wolf p-values (Int. Stress)	0.925	0.925	0.937	0.925	0.925	0.727	0.925	0.925
Romano Wolf p-values (Int. Neg. Emotions)	0.852	0.917	0.949	0.965	0.852	0.378	0.965	0.837
Observations	4,890	4,890	4,890	4,890	4,890	4,890	4,890	4,890
R-squared	0.06	0.08	0.01	0.02	0.01	0.01	0.02	0.02

Note: Robust standard errors in parentheses; *** p<0.01, ** p<0.05, * p<0.1. Romano Wolf p-values correspond to the coefficients of the interactions between shock variable and the recalls. Regressions are done using individual-level control variables in Table S 2.

Table S 12 Difference-in-difference estimation of the effect of negative emotions and stress, using double interaction terms and health shock

VARIABLES	(1) CRT	(2) Risk Taking	(3) Time Discounting	(4) Trust	(5) Altruism 1	(6) Altruism 2	(7) Positive Reciprocity	(8) Negative Reciprocity
Shock # Recall (Stress)	-0.05	0.01	-0.22*	-0.01	-0.01	-0.05	-0.15	0.09
	(0.07)	(0.18)	(0.13)	(0.16)	(0.01)	(0.16)	(0.12)	(0.15)
Shock # Recall (Neg. Emotions)	-0.07	0.10	-0.20	0.01	0.00	-0.13	0.09	-0.09
	(0.07)	(0.18)	(0.13)	(0.16)	(0.01)	(0.16)	(0.12)	(0.15)
Health Shock	-0.12**	0.27**	0.21**	-0.07	0.01	0.15	0.03	0.28***
	(0.05)	(0.13)	(0.09)	(0.12)	(0.01)	(0.12)	(0.08)	(0.11)
Recall (Neg. Emotions)	0.02	-0.16	0.17*	-0.13	-0.00	0.06	-0.03	0.09
	(0.06)	(0.14)	(0.10)	(0.12)	(0.01)	(0.13)	(0.09)	(0.12)
Recall (Stress)	-0.01	-0.08	0.15	-0.16	0.01	-0.09	0.11	0.05
	(0.06)	(0.14)	(0.10)	(0.12)	(0.01)	(0.13)	(0.09)	(0.11)
Age	0.00	-0.03***	0.00	0.02***	0.00	0.01**	0.01***	-0.00*
	(0.00)	(0.00)	(0.00)	(0.00)	(0.00)	(0.00)	(0.00)	(0.00)
Sex	0.24***	0.63***	-0.05	0.05	-0.01	-0.20***	-0.09*	0.28***
	(0.03)	(0.07)	(0.05)	(0.07)	(0.01)	(0.07)	(0.05)	(0.06)
Education	0.10***	0.14***	0.09***	0.11***	0.01***	0.07**	0.04**	0.03
	(0.01)	(0.03)	(0.02)	(0.03)	(0.00)	(0.03)	(0.02)	(0.03)
Income	0.13***	0.11***	0.06*	0.10**	0.00	0.10**	0.07***	0.12***
	(0.02)	(0.04)	(0.03)	(0.04)	(0.00)	(0.04)	(0.03)	(0.04)
Employment	0.05***	-0.17***	-0.05*	-0.05*	0.00	0.05	0.04*	-0.07**
	(0.01)	(0.03)	(0.02)	(0.03)	(0.00)	(0.03)	(0.02)	(0.03)
Residence Space	-0.00**	0.00***	-0.00**	0.00***	-0.00***	0.00***	-0.00*	-0.00***
	(0.00)	(0.00)	(0.00)	(0.00)	(0.00)	(0.00)	(0.00)	(0.00)
Household Size	-0.04***	0.12***	0.04*	0.07***	0.01***	0.06**	0.05**	0.04*
	(0.01)	(0.02)	(0.02)	(0.02)	(0.00)	(0.03)	(0.02)	(0.03)
Constant	-0.29***	4.77***	6.26***	4.40***	0.06***	5.94***	2.70***	5.34***
	(0.10)	(0.27)	(0.20)	(0.25)	(0.02)	(0.25)	(0.18)	(0.23)
Romano Wolf p-values (Int. Stress)	0.945	0.981	0.512	0.981	0.958	0.981	0.801	0.965
Romano Wolf p-values (Int. Neg. Emotions)	0.917	0.979	0.661	0.995	0.995	0.971	0.971	0.979
Observations	4,890	4,890	4,890	4,890	4,890	4,890	4,890	4,890
R-squared	0.06	0.07	0.01	0.02	0.01	0.01	0.02	0.02

Note: Robust standard errors in parentheses; *** p<0.01, ** p<0.05, * p<0.1. Romano Wolf p-values correspond to the coefficients of the interactions between shock variable and the recalls. Regressions are done using individual-level control variables in Table S 2.

Table S 13 Difference-in-difference estimation of the effect of negative emotions and stress, using double interaction terms and stressful events shock

VARIABLES	(1) CRT	(2) Risk Taking	(3) Time Discounting	(4) Trust	(5) Altruism 1	(6) Altruism 2	(7) Positive Reciprocity	(8) Negative Reciprocity
Shock # Recall (Stress)	0.05	-0.08	-0.07	0.04	-0.00	-0.20	-0.05	-0.24
	(0.07)	(0.18)	(0.13)	(0.17)	(0.01)	(0.16)	(0.12)	(0.15)
Shock # Recall (Neg. Emotions)	0.03	-0.18	-0.17	-0.22	-0.01	-0.42**	-0.11	-0.23
	(0.07)	(0.18)	(0.13)	(0.17)	(0.01)	(0.16)	(0.12)	(0.15)
Stressful Events Shock	-0.19***	0.83***	0.29***	0.17	0.01	0.32***	0.09	0.49***
	(0.05)	(0.13)	(0.09)	(0.12)	(0.01)	(0.12)	(0.09)	(0.11)
Recall (Neg. Emotions)	-0.04	0.00	0.15	0.01	0.01	0.23*	0.09	0.17
	(0.06)	(0.14)	(0.10)	(0.13)	(0.01)	(0.13)	(0.09)	(0.12)
Recall (Stress)	-0.07	-0.03	0.06	-0.19	0.00	-0.01	0.06	0.24**
	(0.05)	(0.14)	(0.10)	(0.13)	(0.01)	(0.13)	(0.09)	(0.11)
Age	0.00	-0.02***	0.00*	0.02***	0.00	0.01**	0.01***	-0.00*
	(0.00)	(0.00)	(0.00)	(0.00)	(0.00)	(0.00)	(0.00)	(0.00)
Sex	0.25***	0.62***	-0.06	0.06	-0.01*	-0.21***	-0.09**	0.27***
	(0.03)	(0.07)	(0.05)	(0.07)	(0.01)	(0.07)	(0.05)	(0.06)
Education	0.10***	0.14***	0.09***	0.11***	0.01***	0.07**	0.04**	0.03
	(0.01)	(0.03)	(0.02)	(0.03)	(0.00)	(0.03)	(0.02)	(0.03)
Income	0.12***	0.15***	0.07**	0.11***	0.00	0.10***	0.08***	0.13***
	(0.02)	(0.04)	(0.03)	(0.04)	(0.00)	(0.04)	(0.03)	(0.04)
Employment	0.04***	-0.12***	-0.03	-0.05	0.00	0.05*	0.04**	-0.05*
	(0.01)	(0.03)	(0.02)	(0.03)	(0.00)	(0.03)	(0.02)	(0.03)
Residence Space	-0.00**	0.00***	-0.00**	0.00***	-0.00***	0.00***	-0.00	-0.00***
	(0.00)	(0.00)	(0.00)	(0.00)	(0.00)	(0.00)	(0.00)	(0.00)
Household Size	-0.03***	0.08***	0.03	0.06***	0.01***	0.05**	0.05**	0.03
	(0.01)	(0.02)	(0.02)	(0.02)	(0.00)	(0.03)	(0.02)	(0.03)
Constant	-0.26***	4.27***	6.15***	4.17***	0.06***	5.82***	2.66***	5.21***
	(0.10)	(0.27)	(0.20)	(0.26)	(0.02)	(0.24)	(0.18)	(0.23)
Romano Wolf p-values (Int. Stress)	0.970	0.988	0.988	0.988	0.988	0.789	0.988	0.602
Romano Wolf p-values (Int. Neg. Emotions)	0.772	0.772	0.702	0.702	0.772	0.093	0.772	0.636
Observations	4,890	4,890	4,890	4,890	4,890	4,890	4,890	4,890
R-squared	0.06	0.09	0.01	0.02	0.01	0.01	0.02	0.02

Note: Robust standard errors in parentheses; *** p<0.01, ** p<0.05, * p<0.1. Romano Wolf p-values correspond to the coefficients of the interactions between shock variable and the recalls. Regressions are done using individual-level control variables in Table S 2.

Table S 14 Difference-in-difference estimation of the effect of negative emotions and stress, using double interaction terms and economic vulnerability predicted mental health shock

VARIABLES	(1) CRT	(2) Risk Taking	(3) Time Discounting	(4) Trust	(5) Altruism 1	(6) Altruism 2	(7) Positive Reciprocity	(8) Negative Reciprocity
Shock # Recall (Stress)	-0.09	0.14	-0.17	0.31*	-0.00	0.10	-0.24**	0.02
	(0.06)	(0.17)	(0.13)	(0.16)	(0.01)	(0.16)	(0.11)	(0.15)
Shock # Recall (Neg. Emotions)	-0.08	0.26	-0.05	0.17	0.01	0.06	0.09	0.08
	(0.07)	(0.18)	(0.13)	(0.16)	(0.01)	(0.16)	(0.11)	(0.15)
Economic Vulnerability Predicted Mental Health Shock	-0.12**	0.15	0.02	-0.31***	0.00	0.03	0.01	0.22**
	(0.05)	(0.12)	(0.09)	(0.12)	(0.01)	(0.11)	(0.08)	(0.11)
Recall (Neg. Emotions)	0.01	-0.23*	0.07	-0.22**	-0.00	-0.05	-0.02	-0.00
	(0.05)	(0.13)	(0.09)	(0.11)	(0.01)	(0.11)	(0.08)	(0.10)
Recall (Stress)	-0.00	-0.14	0.10	-0.33***	0.00	-0.17	0.14*	0.10
	(0.05)	(0.12)	(0.09)	(0.11)	(0.01)	(0.11)	(0.08)	(0.10)
Age	0.00	-0.03***	0.00	0.02***	0.00	0.01**	0.01***	-0.00*
	(0.00)	(0.00)	(0.00)	(0.00)	(0.00)	(0.00)	(0.00)	(0.00)
Sex	0.23***	0.64***	-0.06	0.04	-0.01	-0.20***	-0.10**	0.29***
	(0.03)	(0.07)	(0.05)	(0.07)	(0.01)	(0.07)	(0.05)	(0.06)
Education	0.09***	0.15***	0.09***	0.11***	0.01***	0.07**	0.04**	0.03
	(0.01)	(0.03)	(0.02)	(0.03)	(0.00)	(0.03)	(0.02)	(0.03)
Income	0.12***	0.11***	0.05	0.10**	0.00	0.10**	0.07**	0.13***
	(0.02)	(0.04)	(0.03)	(0.04)	(0.00)	(0.04)	(0.03)	(0.04)
Employment	0.05***	-0.17***	-0.05*	-0.05*	0.00	0.05	0.04*	-0.07**
	(0.01)	(0.03)	(0.02)	(0.03)	(0.00)	(0.03)	(0.02)	(0.03)
Residence Space	-0.00**	0.00***	-0.00**	0.00***	-0.00***	0.00***	-0.00*	-0.00***
	(0.00)	(0.00)	(0.00)	(0.00)	(0.00)	(0.00)	(0.00)	(0.00)
Household Size	-0.04***	0.12***	0.04**	0.07***	0.01***	0.06**	0.05***	0.04*
	(0.01)	(0.02)	(0.02)	(0.02)	(0.00)	(0.03)	(0.02)	(0.03)
Constant	-0.27***	4.83***	6.45***	4.59***	0.06***	6.00***	2.74***	5.37***
	(0.10)	(0.27)	(0.20)	(0.25)	(0.02)	(0.25)	(0.18)	(0.23)
Romano Wolf p-values (Int. Stress)	0.634	0.880	0.646	0.327	0.980	0.880	0.256	0.980
Romano Wolf p-values (Int. Neg. Emotions)	0.831	0.698	0.938	0.884	0.938	0.938	0.938	0.938
Observations	4,890	4,890	4,890	4,890	4,890	4,890	4,890	4,890
R-squared	0.06	0.07	0.01	0.02	0.01	0.01	0.02	0.02

Note: Robust standard errors in parentheses; *** p<0.01, ** p<0.05, * p<0.1. Romano Wolf p-values correspond to the coefficients of the interactions between shock variable and the recalls Regressions are done using individual-level control variables in Table S 2.

Table S 15: Effect of shocks on cognitive performance and preferences, using shocks and interaction terms: Italy

		VARIABLE	CRT	Risk Taking	Time Discounting	Trust	Altruism 1	Altruism 2	Positive Reciprocity	Negative Reciprocity
Italy	(1)	Labor Shock	-0.04	0.34**	0.10	0.01	-0.01	-0.04	0.08	0.12
			(0.05)	(0.14)	(0.11)	(0.13)	(0.01)	(0.13)	(0.10)	(0.12)
	(2)	Labor Shock	-0.09	0.50**	0.16	-0.01	-0.00	-0.08	0.17	0.19
			(0.09)	(0.24)	(0.19)	(0.23)	(0.02)	(0.22)	(0.17)	(0.20)
		Shock # Recall	0.06	-0.23	-0.09	0.03	-0.01	0.06	-0.13	-0.09
			(0.11)	(0.29)	(0.22)	(0.27)	(0.02)	(0.27)	(0.20)	(0.25)
	(1)	Health Shock	-0.12**	-0.02	-0.05	-0.10	-0.01	-0.23*	-0.08	0.13
			(0.05)	(0.13)	(0.10)	(0.12)	(0.01)	(0.12)	(0.09)	(0.11)
	(2)	Health Shock	-0.07	-0.13	0.14	-0.20	-0.00	-0.18	-0.04	0.06
			(0.08)	(0.21)	(0.16)	(0.20)	(0.02)	(0.20)	(0.15)	(0.18)
		Shock # Recall	-0.07	0.17	-0.27	0.16	-0.02	-0.06	-0.06	0.10
			(0.10)	(0.26)	(0.19)	(0.25)	(0.02)	(0.25)	(0.18)	(0.22)
	(1)	Stressful Events Shock	-0.13**	0.43***	0.03	0.03	-0.03**	0.08	0.11	0.24**
			(0.06)	(0.15)	(0.11)	(0.14)	(0.01)	(0.13)	(0.10)	(0.12)
	(2)	Stressful Events Shock	-0.18*	0.74***	0.11	0.04	-0.01	0.22	0.25	0.36*
			(0.10)	(0.27)	(0.18)	(0.25)	(0.02)	(0.24)	(0.18)	(0.22)
		Shock # Recall	0.08	-0.44	-0.12	-0.02	-0.03	-0.20	-0.21	-0.17
			(0.12)	(0.31)	(0.22)	(0.29)	(0.02)	(0.29)	(0.21)	(0.26)
	(1)	Economic Vulnerability Predicted Mental Health Shock	-0.09*	-0.04	-0.15	-0.11	-0.01	-0.15	-0.08	0.10
			(0.05)	(0.13)	(0.09)	(0.12)	(0.01)	(0.12)	(0.09)	(0.11)
	(2)	Economic Vulnerability Predicted Mental Health Shock	-0.04	-0.40*	-0.04	-0.45**	-0.00	-0.22	-0.04	-0.03
			(0.08)	(0.21)	(0.16)	(0.20)	(0.02)	(0.20)	(0.15)	(0.18)
		Shock # Recall	-0.07	0.53**	-0.16	0.52**	-0.01	0.09	-0.06	0.20
			(0.10)	(0.26)	(0.19)	(0.25)	(0.02)	(0.24)	(0.18)	(0.22)

Note: Robust standard errors in parentheses; *** p<0.01, ** p<0.05, * p<0.1. (1): Regressions when the main independent variable is shock; (2) Regressions with Shock and an interaction term between Shock and Recall. Regressions are done using individual-level control variables in Table S 2.

:

Table S 16: Effect of shocks on cognitive performance and preferences, using shocks and interaction terms: UK

		VARIABLE	CRT	Risk Taking	Time Discounting	Trust	Altruism 1	Altruism 2	Positive Reciprocity	Negative Reciprocity
UK	(1)	Labor Shock	-0.13**	0.69***	0.07	0.17	-0.01	0.21*	0.00	-0.02
			(0.05)	(0.13)	(0.10)	(0.12)	(0.01)	(0.12)	(0.09)	(0.11)
	(2)	Labor Shock	-0.18**	0.79***	0.20	0.24	-0.01	0.40**	-0.12	-0.05
			(0.09)	(0.22)	(0.16)	(0.21)	(0.02)	(0.20)	(0.15)	(0.19)
		Shock # Recall	0.07	-0.15	-0.20	-0.10	0.01	-0.29	0.19	0.04
			(0.10)	(0.26)	(0.20)	(0.25)	(0.02)	(0.24)	(0.17)	(0.22)
	(1)	Health Shock	-0.20***	0.50***	0.13	-0.15	0.02*	0.32***	0.15*	0.47***
			(0.05)	(0.14)	(0.10)	(0.12)	(0.01)	(0.12)	(0.09)	(0.11)
	(2)	Health Shock	-0.17*	0.42*	0.25	-0.09	0.01	0.53***	0.20	0.54***
			(0.09)	(0.22)	(0.16)	(0.21)	(0.02)	(0.20)	(0.14)	(0.18)
		Shock # Recall	-0.05	0.11	-0.20	-0.10	0.01	-0.33	-0.08	-0.11
			(0.11)	(0.26)	(0.20)	(0.25)	(0.02)	(0.24)	(0.17)	(0.22)
	(1)	Stressful Events Shock	-0.24***	0.74***	0.14	0.17	0.03***	0.23*	0.02	0.28**
			(0.05)	(0.14)	(0.10)	(0.13)	(0.01)	(0.12)	(0.09)	(0.12)
	(2)	Stressful Events Shock	-0.19**	0.58**	0.33*	0.30	0.03*	0.60***	0.12	0.36*
			(0.09)	(0.23)	(0.17)	(0.22)	(0.02)	(0.21)	(0.15)	(0.20)
		Shock # Recall	-0.08	0.23	-0.30	-0.19	-0.01	-0.56**	-0.15	-0.11
			(0.11)	(0.27)	(0.20)	(0.26)	(0.02)	(0.25)	(0.18)	(0.23)
	(1)	Economic Vulnerability Predicted Mental Health Shock	-0.24***	0.48***	-0.08	-0.33**	0.02**	0.33***	0.18**	0.45***
			(0.05)	(0.14)	(0.10)	(0.13)	(0.01)	(0.13)	(0.09)	(0.12)
	(2)	Economic Vulnerability Predicted Mental Health Shock	-0.20**	0.40*	0.09	-0.29	0.02	0.52**	0.37***	0.47**
			(0.09)	(0.22)	(0.17)	(0.22)	(0.02)	(0.20)	(0.14)	(0.19)
		Shock # Recall	-0.06	0.13	-0.25	-0.07	0.00	-0.30	-0.29*	-0.04
			(0.10)	(0.26)	(0.20)	(0.25)	(0.02)	(0.24)	(0.17)	(0.23)

Note: Robust standard errors in parentheses; *** p<0.01, ** p<0.05, * p<0.1. (1): Regressions when the main independent variable is shock; (2) Regressions with Shock and an interaction term between Shock and Recall. Regressions are done using individual-level control variables in Table S 2.

Table S 17. Effect of shocks on cognitive performance and preferences, using shocks and interaction terms: Spain

		VARIABLE	CRT	Risk Taking	Time Discounting	Trust	Altruism 1	Altruism 2	Positive Reciprocity	Negative Reciprocity
Spain	(1)	Labor Shock	-0.09**	0.42***	0.02	0.05	0.00	0.26**	0.08	0.21*
			(0.05)	(0.13)	(0.09)	(0.12)	(0.01)	(0.12)	(0.08)	(0.11)
	(2)	Labor Shock	-0.09	0.27	-0.16	-0.14	0.01	0.10	0.04	0.44**
			(0.08)	(0.21)	(0.15)	(0.19)	(0.02)	(0.19)	(0.14)	(0.19)
		Shock # Recall	-0.00	0.23	0.27	0.29	-0.00	0.23	0.06	-0.34
			(0.10)	(0.26)	(0.19)	(0.24)	(0.02)	(0.23)	(0.17)	(0.23)
	(1)	Health Shock	-0.10**	0.51***	0.18*	-0.14	0.01	0.25**	-0.09	0.29**
			(0.05)	(0.14)	(0.10)	(0.12)	(0.01)	(0.12)	(0.09)	(0.12)
	(2)	Health Shock	-0.05	0.40*	0.17	-0.12	0.01	0.12	-0.22	0.10
			(0.08)	(0.22)	(0.16)	(0.20)	(0.02)	(0.20)	(0.14)	(0.19)
		Shock # Recall	-0.07	0.17	0.02	-0.03	-0.00	0.19	0.19	0.28
			(0.10)	(0.28)	(0.20)	(0.25)	(0.02)	(0.25)	(0.18)	(0.24)
	(1)	Stressful Events Shock	-0.09*	0.75***	0.21**	0.28**	0.00	0.17	-0.18**	0.30**
			(0.05)	(0.13)	(0.10)	(0.13)	(0.01)	(0.12)	(0.09)	(0.12)
	(2)	Stressful Events Shock	-0.15*	0.68***	0.14	0.19	-0.01	0.19	-0.26*	0.49**
			(0.08)	(0.22)	(0.15)	(0.20)	(0.02)	(0.20)	(0.14)	(0.19)
		Shock # Recall	0.09	0.10	0.11	0.15	0.02	-0.02	0.12	-0.28
			(0.10)	(0.27)	(0.19)	(0.25)	(0.02)	(0.24)	(0.17)	(0.23)
	(1)	Economic Vulnerability Predicted Mental Health Shock	-0.17***	0.38***	0.06	-0.10	-0.00	0.12	-0.23***	0.22**
			(0.05)	(0.13)	(0.10)	(0.12)	(0.01)	(0.11)	(0.08)	(0.11)
	(2)	Economic Vulnerability Predicted Mental Health Shock	-0.05	0.26	-0.06	-0.34*	-0.01	-0.23	-0.35***	0.13
			(0.08)	(0.21)	(0.16)	(0.19)	(0.02)	(0.18)	(0.13)	(0.19)
		Shock # Recall	-0.18*	0.18	0.18	0.35	0.02	0.52**	0.18	0.14
			(0.09)	(0.26)	(0.19)	(0.23)	(0.02)	(0.23)	(0.16)	(0.22)

Note: Robust standard errors in parentheses; *** p<0.01, ** p<0.05, * p<0.1. (1): Regressions when the main independent variable is shock; (2) Regressions with Shock and an interaction term between Shock and Recall. Regressions are done using individual-level control variables in Table S 2.
